# Surrogate modeling of passive microwave circuits using recurrent neural networks and domain confinement

**DOI:** 10.1038/s41598-025-91643-3

**Published:** 2025-04-17

**Authors:** Kaustab C. Sahu, Slawomir Koziel, Anna Pietrenko-Dabrowska

**Affiliations:** 1 Engineering Optimization & Modeling Center, Reykjavik University, 101 Reykjavik, Iceland; 2 Faculty of Electronics, Telecommunications and Informatics, Gdansk University of Technology, 80-233 Gdansk, Poland

**Keywords:** Microwave circuits, Data-driven surrogates, Recurrent neural networks, Sensitivity analysis, Domain confinement, Engineering, Electrical and electronic engineering

## Abstract

Electromagnetic (EM) simulation is widespread in microwave engineering. EM tools ensure evaluation reliability but incur significant expenses. These can be mitigated by employing surrogate modeling methods, especially to expedite design workflows like local/global optimization or uncertainty quantification. However, building accurate surrogates is a daunting task beyond simple cases (low dimensionality, narrow geometry parameter and frequency ranges). This research suggests a new technique for dependable modeling of microwave circuits. Its main ingredient is a recurrent neural network (RNN) with the main architectural components being bidirectional Long Short-Term Memory (LSTM) and Gated Recurrent Unit (GRU) layers. These are incorporated to accurately represent frequency relationship within circuit characteristics as well as dependencies between its dimensions and outputs considered as vector-valued functions parameterized by frequency. The network’s hyperparameters are adjusted through Bayesian Optimization (BO). Utilization of frequency as a sequential variable handled by RNN is a distinguishing feature of our approach, which leads to the enhancement of dependability and cost efficiency. Another critical factor is dimension- and volume-wise reduction of the model’s domain achieved through global sensitivity analysis. It allows for additional and dramatic accuracy improvements without diminishing the surrogate’s coverage regarding circuit’s operating parameters. Our methodology has been extensively validated using several microstrip structures. The results demonstrate its competitive performance over a range of kernel-based regression techniques and diverse neural networks. The proposed procedure ensures building models of outstanding predictive power while using small training datasets, which is beyond the capabilities of benchmark algorithms.

## Introduction

Computational tools are indispensable in modern microwave engineering^1,2^ with the special emphasis on electromagnetic (EM) solvers^3,4^. EM simulation is versatile and enables quantification of effects that cannot be evaluated using different methods (cross-coupling, dielectric/radiation losses, anisotropy, the impact of environmental components such as connectors or installation fixtures). For many circuits, such as compact structures, substrate-integrated waveguide (SIW)-based circuits, or structures incorporating metamaterials^5–9^, EM simulation is imperative when it comes to accurate characterization of their electrical properties. However, EM analysis is CPU intensive, which impedes its utilization in procedures requiring multiple system evaluations. Examples include design closure^10,11^, statistical analysis^12–14^, or global and multi-objective design^15–18^. The latter seems to be the most challenging endeavor, typically executed using bio-inspired algorithms with expenses reaching thousands of merit function calls^19–23^.

Recent years observed significant research focus on expedited EM-driven design methodologies. A range of methods were developed to accelerate gradient-based algorithms (adjoint sensitivity, restricted Jacobian updating, parallelization, mesh deformation^24–28^). More generic approaches include feature-based techniques^29,30^, variable-fidelity approaches^31–34^, or dimensionality reduction^35–38^. Notwithstanding, the main emphasis is currently put on surrogate-assisted procedures^39–43^, typically arranged as machine learning (ML) algorithms^44–46^. Widely used modelling methods include support vector regression, Gaussian processes, kriging, polynomial chaos expansion, ensemble learning, and diverse types of neural networks^47–55^.

Shifting computational operations to a fast metamodel effectively mitigates the cost-related issues of EM-driven design. At the same time, constructing reliable surrogates is a difficult undertaking except for simple scenarios (small number of design variables, narrow parameter ranges). The main problem is the curse of dimensionality^56^ and the large volume of the traditional (interval-based) domain. Both factors lead to a fast increase in the size of the training set necessary to build an accurate data-driven model. This is why surrogate-based procedures normally take the form of ML algorithms in which case the surrogate is constructed in a restricted region believed to encapsulate the optimum design. Much of the space is not explored. Clearly, data-driven models constructed during the ML-based search are not general-purpose^57^. Method for alleviating the mentioned difficulties in a more generic setting include better utilization of available data (ensemble learning^58,59^), improved handling of large datasets (deep learning^60,61^), exploring specific system output structure (high-dimensional model representation, HDMR, orthogonal matching pursuit^62,63^). Alternative techniques include multi-fidelity methods^64–66^, and performance-driven modelling^67–71^. Therein, the domain is limited to a subspace containing high-quality designs. Approximation of this region requires extra computational effort^67^; however, the surrogate exhibits predictive power that cannot be matched by conventional methods.

Recently, the growing popularity of artificial neural networks (ANNs) has been observed in modelling of high-frequency structures. ANNs are typically used within ML-based design procedures. The employed architectures are often variations of feedforward networks, specifically multi-layer perceptrons (MLPs)^72–78^, convolutional neural networks (CNNs)^79–81^, deep neural networks (DNNs)^82–84^. At times, specialized architectures are applied, e.g., long short-term memory (LSTM) layers^85^, multi-fidelity^86^, evolutionary^87^, autoencoders^88^). Some studies use inverse ANNs^89,90^. General-purpose ANN-based modeling is rare due to challenges discussed earlier. Some exemplary works involve DNN^53^, CNN^91^, MLP^92^, graph neural networks^93^, cascaded networks^94^, sensitivity networks^95^, or hybrid networks^96^. The mentioned works utilize ANNs as regressors without exploring intricate relationships between decision variables and system features encoded in frequency responses. As a result, their operation and reliability are similar to conventional regression techniques.

This paper introduces an innovative strategy for high-performance surrogate modelling of microwave circuits. The proposed method leverages handling *S*-parameter characteristics as sequential data ensembles parameterized by frequency and exploring their dependence on decision variables (e.g., geometry parameters). The underlying surrogate is a recurrent neural network (RNN). RNNs are well-suited to processing sequential information. The specific RNN architecture incorporates single- and bi-directional Long Short-Term Memory (LSTM) layers, the Gated Recurrent Unit (GRU) layer, and fully connected and (output) regression layers to produce the final predictions of the complete frequency responses. The model’s hyperparameters, including the number of units in each layer, are adjusted through Bayesian Optimization (BO). Treating frequency as a sequential parameter distinguishes our approach from conventional regression and machine learning methods while being advantageous for the dependability and overall efficacy of the modeling process. Dimensionality reduction realized with global sensitivity analysis (GSA) constitutes another mechanism incorporated to improve the surrogate’s accuracy dramatically. GSA aims to yield orthogonal directions responsible for the maximum variability of the system at hand and span the restricted domain along them. The modeling strategy developed in this work is validated with the help of several microstrip circuits and compared to a range of benchmark techniques such as neural networks and diverse regression surrogates. The results underscore the remarkable predictive power of our metamodels, which is superior to all benchmark techniques. Considerable improvement is observed regardless of whether the model is established in the conventional (box-constrained) domain or dimensionality-reduced region. Furthermore, usable surrogates can be constructed using small numbers of training points, which was not the case for most of the comparison methods.

The original contributions of this study include (i) the development of a novel RNN-based metamodel for precise representing of circuit’s characteristics, (ii) handling frequency as a sequential parameter to facilitate the data-driven representation of the scattering parameters and capture the relationships between systems outputs at various frequencies and the design variables, (iii) incorporating LSTM and GRU layers for efficiency frequency-wise dependencies processing, (iv) utilization of Bayesian optimization for boosting the surrogate’s accuracy, (v) development of complete modeling framework that leverages explicit dimensionality reduction through global sensitivity analysis, (vi) demonstrating remarkable reliability of our method and its advantages over several benchmark procedures.

## RNN and dimensionality reduction for precise microwave circuit modeling

The fundamental components of the presented modeling approach are elaborated here. We first recall the formulation of the modeling problem in Sect. “Microwave modelling”. The overall structure and the working principles of the suggested RNN are provided in Sect. “RNN-based surrogates with sequential processing of frequency responses”. The domain confinement mechanism is discussed in Sect. “Domain Confinement Using Global Sensitivity Analysis”, whereas Sect. “Modeling Procedure” puts together the operating flow of the complete algorithm.

### Microwave modelling

Let ***R***_*f*_(***x***) represent the primary (EM-simulated) model of the circuit of interest. Here, ***x*** = [*x*_1_ … *x*_*n*_]^*T*^ are design parameters. ***R***_*f*_ stands for the aggregated system outputs, typically scattering parameters *S*_*kj*_(***x***,*f*), where *f* is frequency, and *k* and *j* mark the respective circuit ports. We aim to build a low-cost surrogate ***R***_*s*_(***x***) that accurately represents ***R***_*f*_(***x***) within the domain *X*. Traditionally, *X* is an interval [***l u***] defined by the lower and upper bounds for parameters ***l*** = [*l*_1_ … *l*_*n*_]^*T*^ and ***u*** = [*u*_1_ … *u*_*n*_]^*T*^.

The surrogate’s accuracy is evaluated by means of a suitable error metric (see, e.g.,^97,98^). Here, the relative root-mean-square error (RRMSE) is used, defined as ||***R***_*s*_(***x***) – ***R***_*f*_(***x***)||/||***R***_*f*_(***x***)|| (if system responses contain multiple vectors, the Frobenius norm is employed). To estimate the accuracy over the entire domain, the average error *E*_*aver*_ is computed.1

where {**x**_t_^(k)^}_k = 1, …, Nt_, are independent testing (hold-off) samples. The relative error is convenient as it matches well the visual alignment between the metamodel and EM evaluated outputs. Less than ten percent of RRMSE typically translates into good alignment between **R**_f_ and **R**_s_ and makes the model suitable for design purposes.

### RNN-based surrogates with sequential processing of frequency responses

This section describes the architecture of the proposed recurrent neural network (RNN) surrogate with sequential frequency data processing. The underlying concept is elucidated in Sect. “RNN modeling with sequential frequency processing”. Section “Model Structure” discusses the network structure, essential layers, and the working principles. RNN training and hyperparameter optimization are outlined in Sect. “Hyperparameter Optimization”.

#### RNN modeling with sequential frequency processing

Predicting the frequency characteristics of microwave signals poses a unique challenge. However, the modeling process may be facilitated by exploring dependencies between the signal state at the given frequency and the lower and higher ones. Thus, instead of traditional methods, Recurrent Neural Networks, which are designed to recognize patterns over sequences, are well-suited for this purpose. The central feature of an RNN is its ability to retain information across time steps through a recurrent structure, allowing it to capture dependencies over extended sequences. When applied to frequency data, RNNs treat each frequency point as a sequential input, which allows the model to learn from the dependencies between these points.

Frequency data often exhibits dependencies that vary across scales—both in the immediate range (e.g., a particular shape of a resonance) and over more extended periods (e.g., relationship between the fundamental operating frequency and the harmonics), necessitating a model that can remember and adjust based on both recent and long-past data. Thus, to account for the same the current model combines several types of RNN layers with optimization strategies such that the predicted value is as close to corresponding frequency characteristic.

#### Model structure

**Fig. 1:**
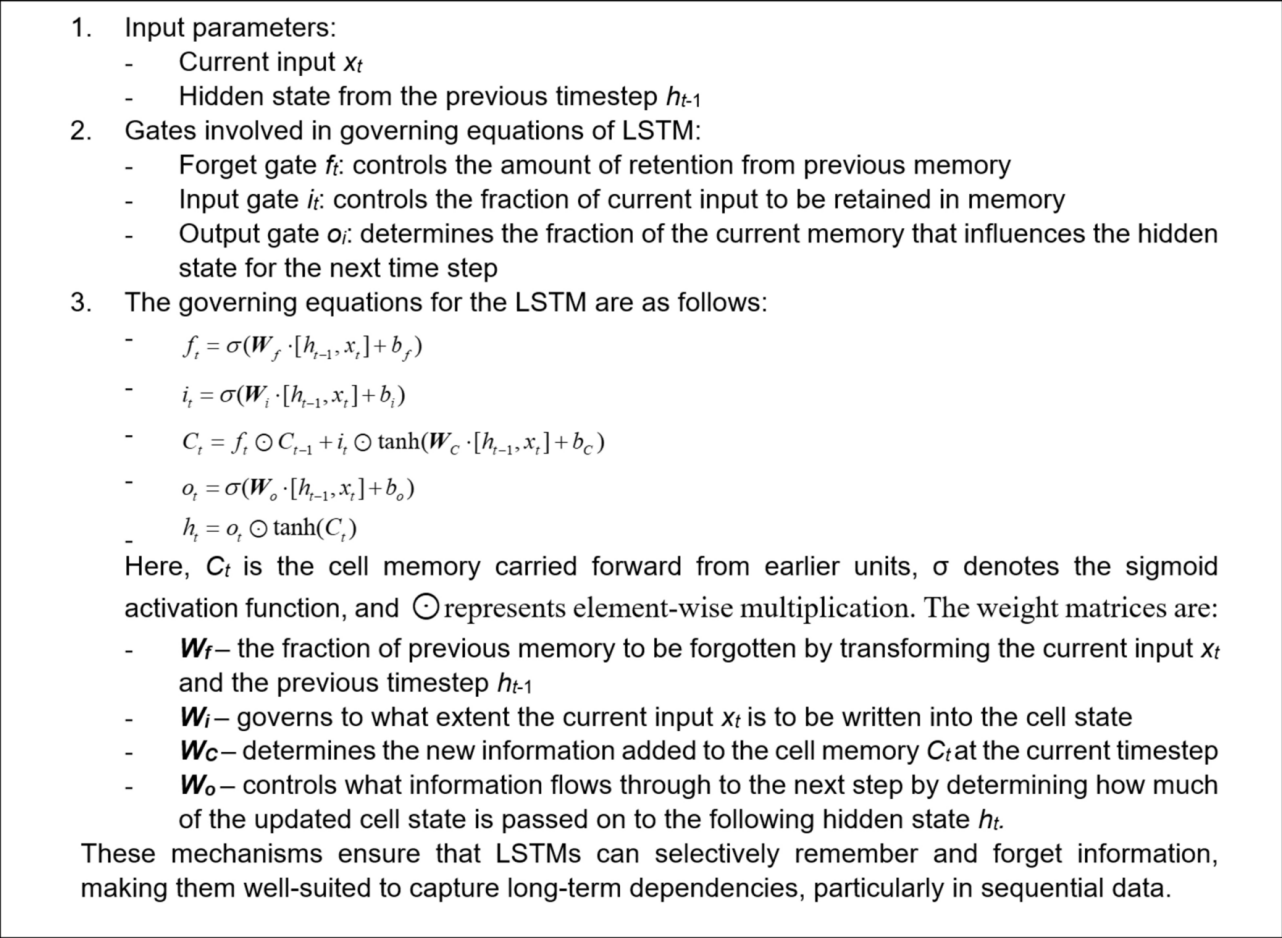
LSTM and its governing equations. This layer is designed to capture long-term dependencies in sequential data. In this case the gated mechanisms regulate the flow of information, selectively remembering or forgetting past inputs to model complex temporal relationships over extended sequences.

**Fig. 2:**
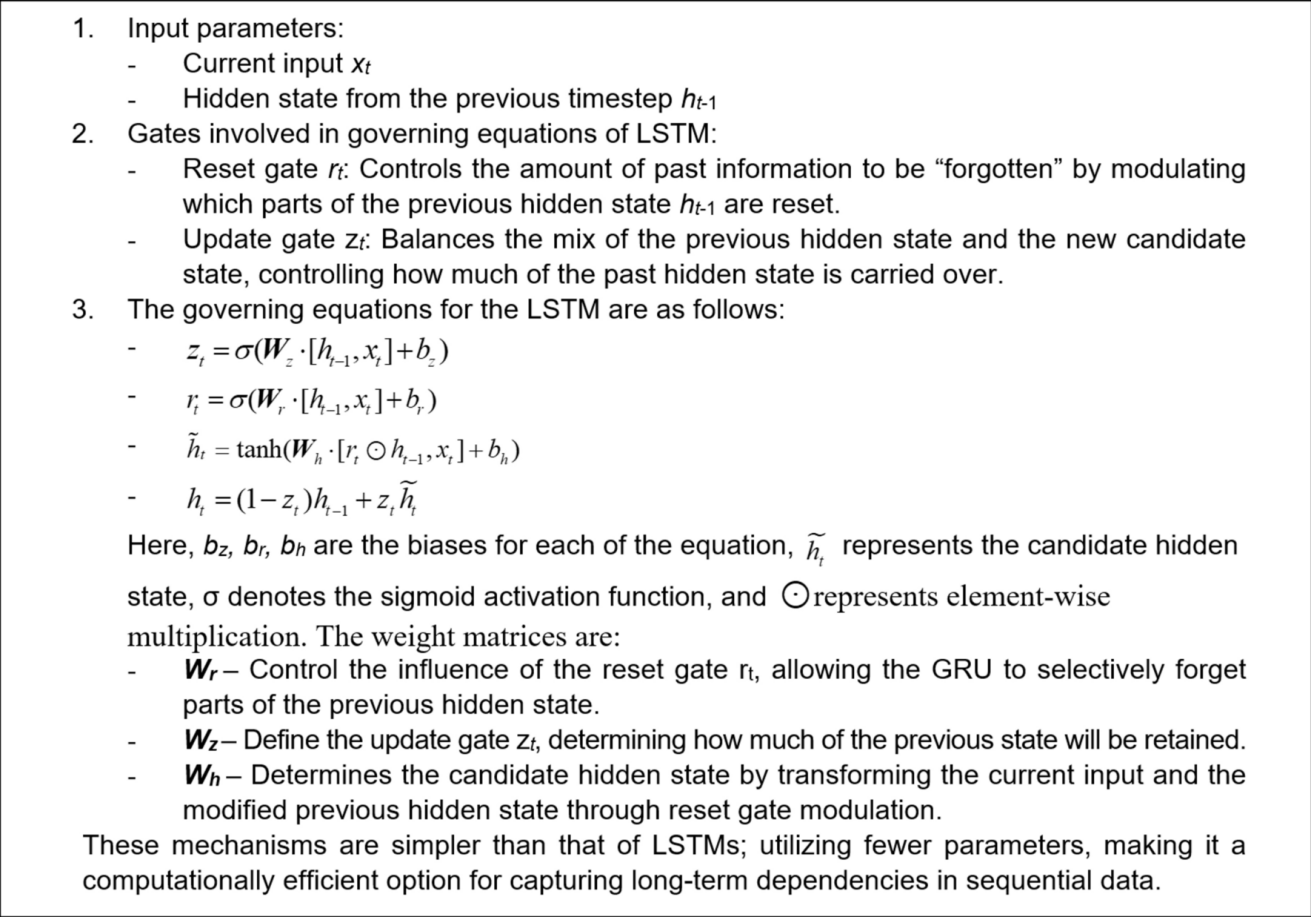
GRU and its governing equations. A streamlined variant of the LSTM, designed to reduce computational complexity while retaining the ability to capture long-term dependencies in sequential data.

**Fig. 3:**
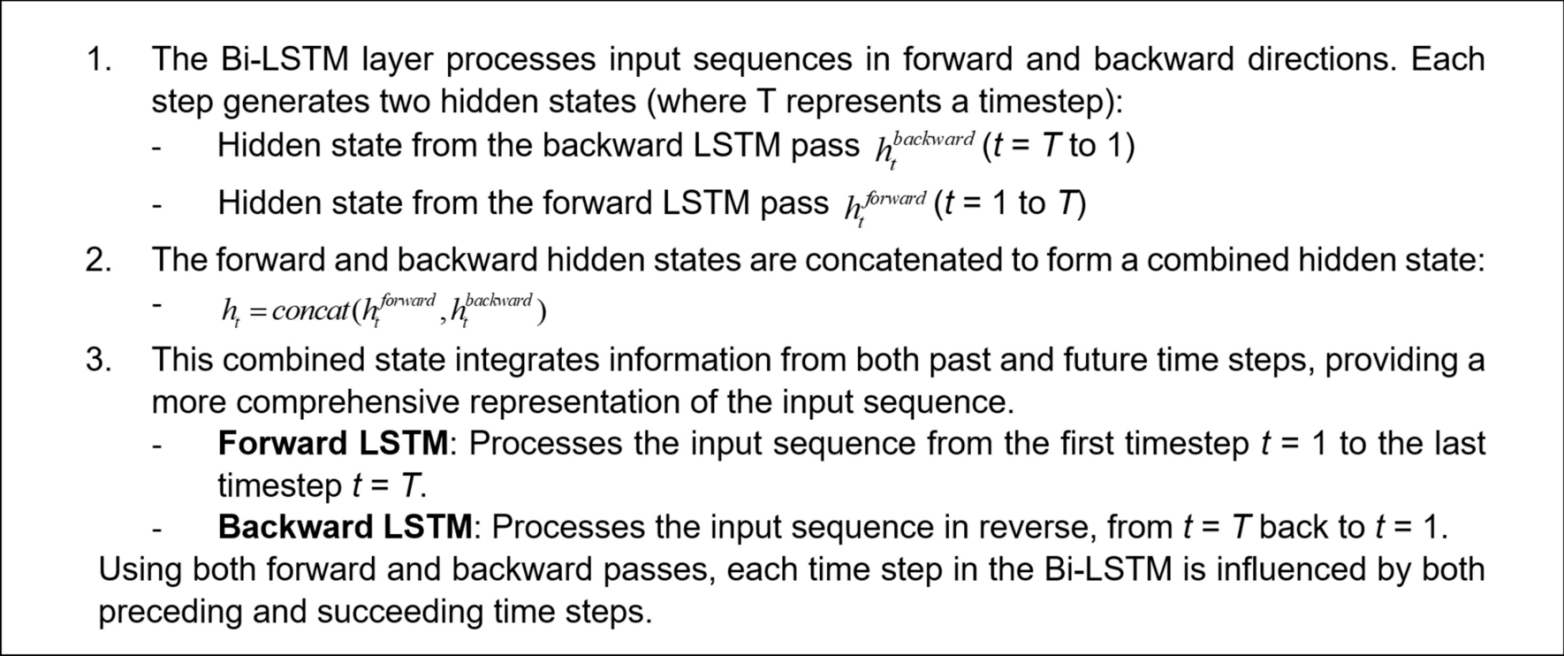
Bi-LSTM and its governing equations. The bidirectional approach enables the model to capture temporal relationships more completely, enhancing its understanding of sequential data.

**Fig. 4:**
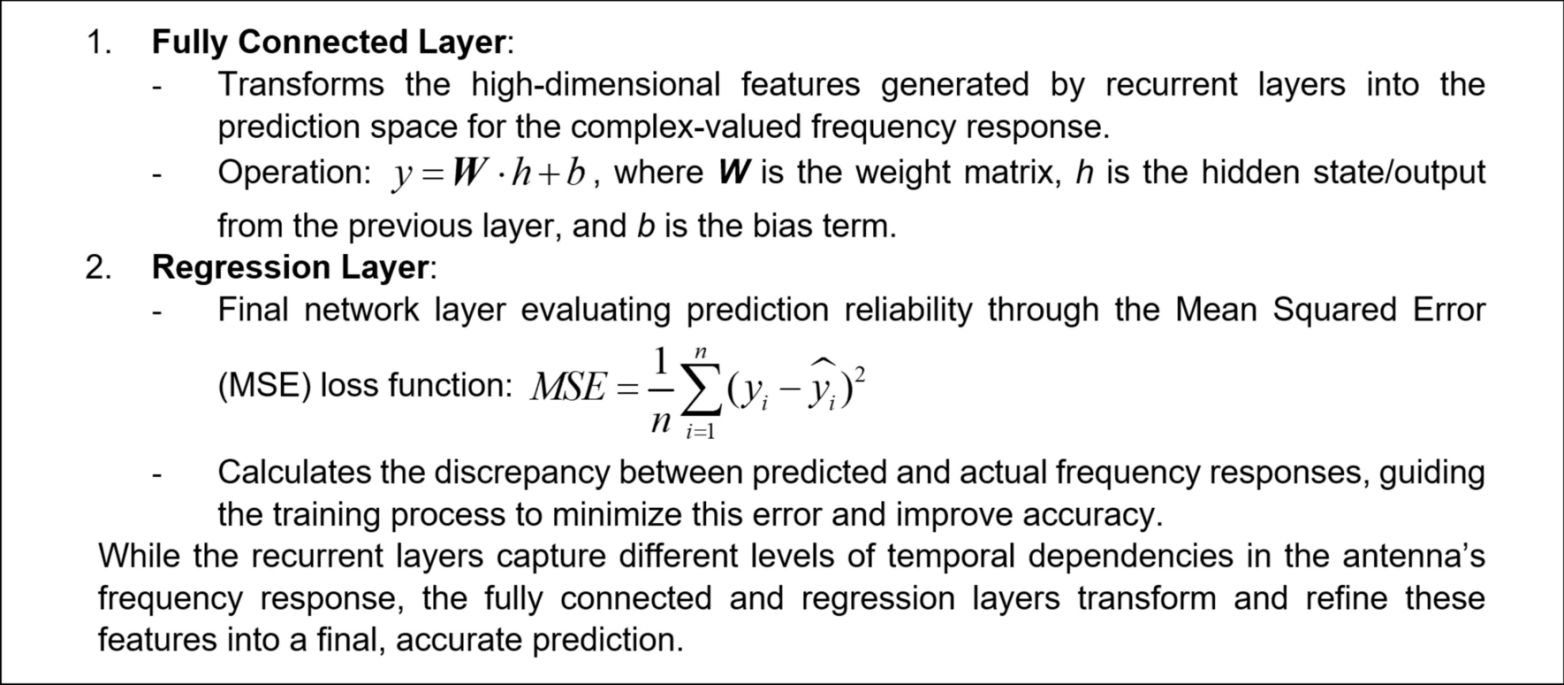
Fully connected layer. It is the final layer of the proposed model. It maps the high-dimensional features from recurrent layers into a prediction space for complex-valued responses. In contrast, the regression layer minimizes prediction error by assessing the discrepancy with actual responses. Together, these layers refine the output for improved prediction accuracy.

For an effective learning and accurate frequency response prediction, the proposed model combines a set of LSTM, GRU and Bi-LSTM layers. In this section, we introduce and explore the specific roles of each of the layers. The operating principles of the LSTM layer have been shown in Fig. 1. The purpose of this layer is to capture long-term dependencies in the sequential data. The gated mechanisms regulate the flow of information, selectively remembering or forgetting past inputs to model complex temporal relationships over extended sequences. The second type is a GRU layer (cf. Figure 2), which is a streamlined variant of the LSTM. GRU is designed to reduce computational complexity while retaining the ability to representing sequential data dependencies, which, in our case, are the relationships between the circuit’s response along the considered frequency spectrum. Yet another tool incorporated into the proposed model is the bidirectional LSTM (Bi-LSTM) layer, shown in Fig. 3, which enables the model to capture temporal relationships in a more comprehensive manner by making the system representation at any given frequency dependent on both lower and higher parts of the spectrum. Fully connected and regression layers (Fig. 4) are the final components of the proposed RNN surrogate.

**Fig. 5:**
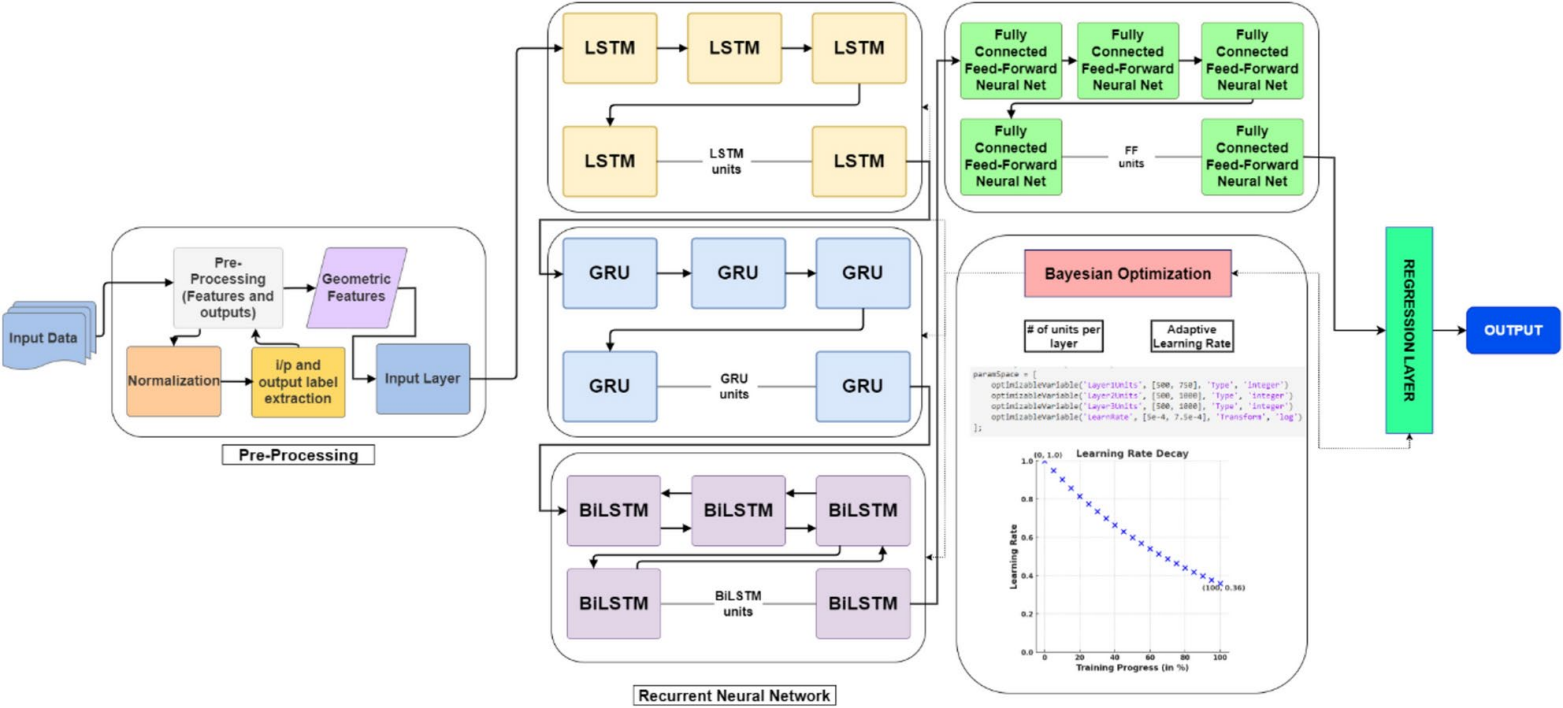
Architecture diagram of the proposed model.

Their role of the former is to map the high-dimensional features from recurrent layers into a prediction space for rendering complex-valued responses. The latter minimizes prediction error by assessing the discrepancy with actual responses. Together, these layers refine the output for improved prediction accuracy. The architecture of the overall RNN metamodel can be found in Fig. 5. The input features *x* and the output signals *y* are extracted and structured in the pre-processing module. The RNN processes the input features through a sequence of layers, starting with the LSTM layer followed by GRU to capture long-term dependencies, and Bi-LSTM layers for efficient modeling and bidirectional context to build the microwave outputs. These recurrent layers extract temporal features, which are then transformed by fully connected layers into prediction space. Finally, the regression layer evaluates the model by comparing the predicted complex-valued frequency responses to the actual values using the relative error metric.

Initial experimentation with different configurations of LSTM and GRU layers revealed challenges in achieving an optimal balance between underfitting and overfitting. Models using only LSTM or GRU layers struggled to capture the full complexity of the data, but with increased layer depth, it exhibited signs of overfitting. Similarly, a fully Bi-LSTM-based architecture, while providing bidirectional context, resulted in excessive training times without substantial gains in accuracy. Adjusting the number of neurons per layer in these configurations did not yield improvements beyond existing benchmarks. Following multiple rounds of testing, we determined that a hybrid architecture combining LSTM, GRU, and Bi-LSTM layers provided the best trade-off between accuracy, generalization, and training efficiency. Specifically, the LSTM layers capture long-term dependencies in the sequence, GRU layers enable deeper architectures with reduced computational cost compared to LSTM, and Bi-LSTM layers enhance contextual understanding by incorporating bidirectional dependencies. This architecture was finalized after systematically evaluating alternative designs and observing consistent performance gains across different datasets (see Sect. “Modeling Procedure”).

#### Hyperparameter optimization

**Fig. 6:**
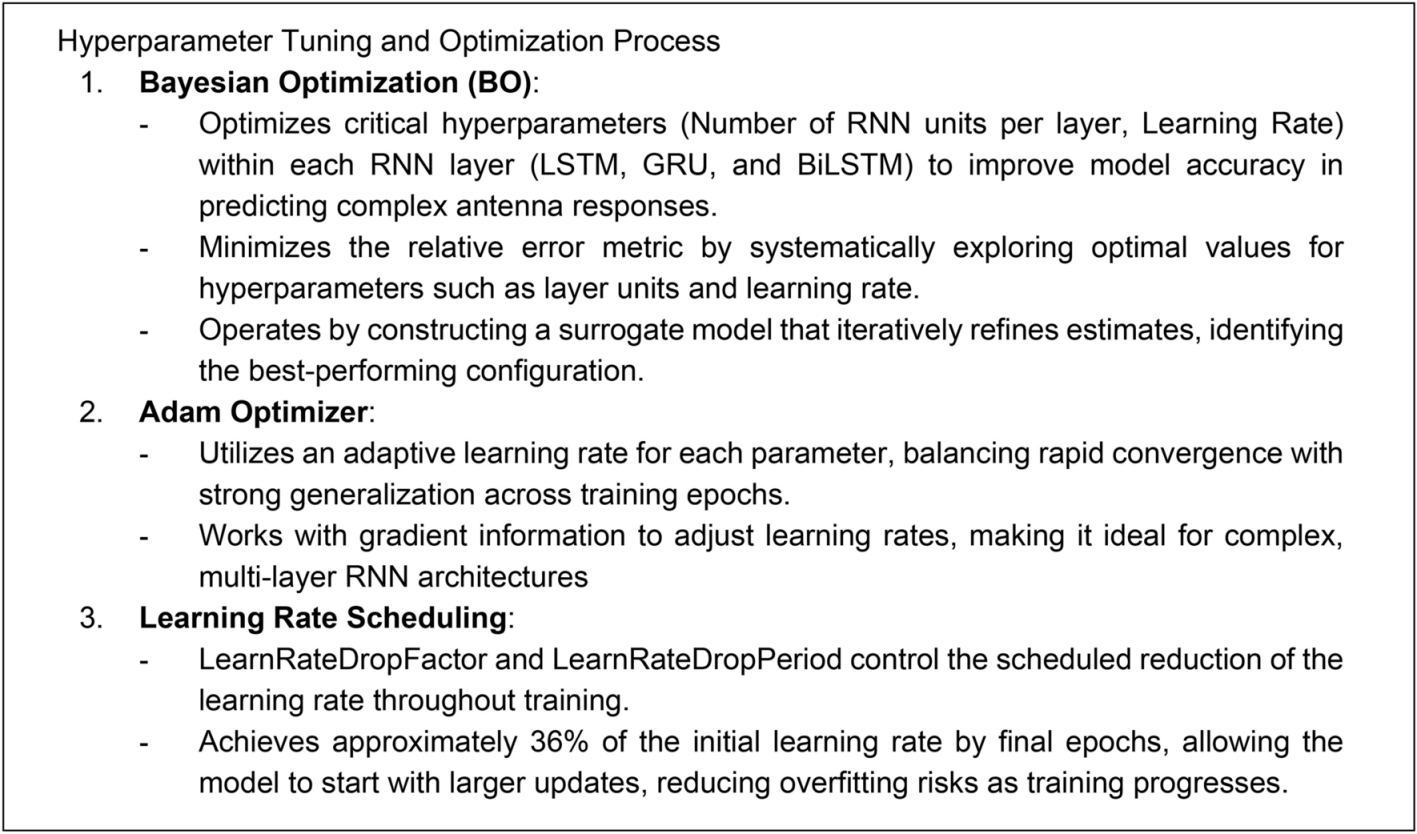
Optimization strategy for RNN model training. Bayesian Optimization^99^ tunes essential hyperparameters to balance model complexity and generalization, while the Adam optimizer^100^ and controlled learning rate adjustments ensure effective convergence without overfitting.

The hyperparameter tuning and optimization process is essential for refining the model’s predictive capability, accuracy, and generalization. In this work, it is realized using Bayesian Optimization (BO)^99^ at the level of the number of RNN units per layer, learning rate, etc. Its purpose is to identify the best possible model architecture. At the same time, the layer-specific parameters, i.e., the weights, are optimized using the ADAM procedure^100^. Figure 6 summarizes the operations undertaken to identify the model and the roles of the applied algorithmic tools.

### Domain confinement using global sensitivity analysis

Appropriate handling of the training data is one of the essential aspects of reliable surrogate modeling. The second is the proper selection of the model domain, which—in this study—is based on dimensionality reduction involving fast global sensitivity analysis (FGSA), initially proposed in^69^. The idea is to span the domain along the vectors responsible for the most significant changes in the system outputs while leaving out the remaining directions.

**Fig. 7:**
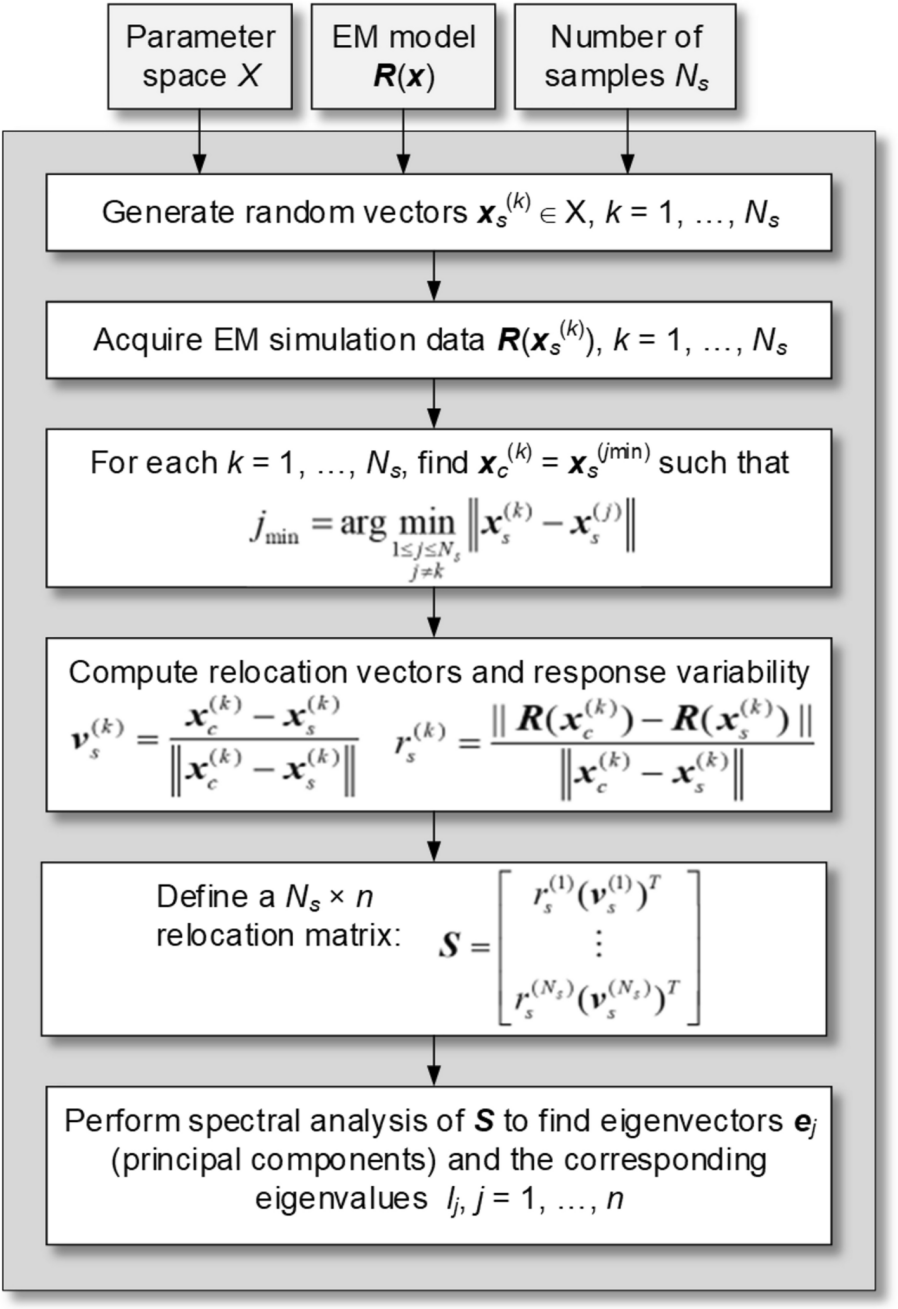
The outline of FGSA^69^. The vectors ***e***_*j*_ account for the directions that significantly affect the system outputs; their relevance is assessed by the eigenvalues λ_*j*_.

This enables the dimensionality-related difficulties to be tackled without compromising the design utility of the model. FGSA is used instead of conventional techniques such as variable screening^101–103^ or traditional global sensitivity analysis (Sobol indices, Jansen’s method^104–106^) capitalizing on its low running cost and flexibility. In particular, the essential directions constructed by FGSA can be arbitrarily oriented (i.e., not aligned with coordinate system axes), which enables accounting for joint effects of pairs or triples of design variables without eliminating individual parameters. The operating flow of FGSA has been outlined in Fig. 7. The random vectors ***x***_*s*_^(*k*)^ are generated using Latin Hypercube Sampling (LHS)^107^. The eigenvectors and eigenvalues are found using principal component analysis^108^. The eigenvalues are arranged in descending order λ_1_ ≥ λ_2_ ≥ … ≥ λ_*n*_, i.e., the effect of subsequent eigenvectors on the circuit’s characteristics gradually decreases.

The dimensionality-reduced domain of the metamodel is spanned by *N*_*d*_ most critical eigenvectors ***e***_*j*_, which collectively account for most of the system’s response variability. Let *C*_min_ be the joint variability threshold. The number *N*_*d*_ is the smallest integer such that^99^

2 $${{\sqrt {\sum\nolimits_{j = 1}^{{N_{d} }} {\lambda_{j}^{2} } } } \mathord{\left/ {\vphantom {{\sqrt {\sum\nolimits_{j = 1}^{{N_{d} }} {\lambda_{j}^{2} } } } {\sqrt {\sum\nolimits_{j = 1}^{n} {\lambda_{j}^{2} } } }}} \right. \kern-0pt} {\sqrt {\sum\nolimits_{j = 1}^{n} {\lambda_{j}^{2} } } }} \ge C_{\min }$$

In this work, *C*_min_ = 0.9 so that the vectors ***e***_*j*_ defining the domain are associated with 90% of the circuit’s response variability.

The reduced domain *X*_*d*_ is defined as^69^3

where x_c_ = [l + u]/2 is the original domain’s center, whereas a_j_, j = 1, …, N_d_, are real numbers. In other words, X_d_ is an intersection of the N_d_-dimensional subspace determined by e_j_, j = 1, …, N_d_, and the conventional parameter space X.

### Modeling procedure

This section puts together the operation of the complete modeling framework utilizing the components elucidated in Sects. “Microwave Modelling” through Sects. “Domain Confinement Using Global Sensitivity Analysis”. The focus is on the training process and model validation.

#### Data extraction and preprocessing

Data extraction is a critical first step in preparing the input and output variables for RNN training. The dataset consists of four sets of complex-valued frequency responses, and through the pre-processing module in Fig. 5, we structure the data into matrices that represent the antenna’s behavior across frequencies, formatted for sequential processing by the RNN.

#### Model architecture and design

As mentioned earlier, the proposed architecture integrates multiple types of recurrent layers, each selected for its unique strengths in processing temporal dependencies. The first part of the model is an input layer that feeds into an LSTM (Long Short-Term Memory) layer. LSTM captures long-term dependencies within the frequency sequence by retaining past information across time steps. Following the LSTM layer, a GRU (Gated Recurrent Unit) layer is added to provide efficient processing with fewer parameters, maintaining relevant patterns in the sequence without excessive computational overhead. Subsequently, a Bi-LSTM (Bidirectional LSTM) layer is incorporated to process the sequence in both directions (forward and backward), which enables the model to utilize contextual information from both preceding and succeeding frequency points. This bidirectional setup enhances the model’s understanding of the sequence, as it learns from both past and future data points (here, meaning signal levels corresponding to the lower and higher frequencies) simultaneously.

The outputs from these recurrent layers are processed by a fully connected layer, which performs a linear transformation that maps the high-dimensional feature space into the final prediction space, representing the complex-valued frequency response of the antenna. Finally, a regression layer computes the model’s prediction quality using the Mean Squared Error (MSE) as the loss function. This layer guides the training process by iteratively adjusting the model parameters to minimize prediction error. The combination of these recurrent layers with optimized hyperparameters, forms a robust architecture capable of capturing both short-term and long-term dependencies in the antenna’s frequency response, resulting in highly accurate and generalizable predictions.

#### Hyperparameter tuning

To optimize the model’s accuracy, Bayesian Optimization (BO) is employed as mentioned in Sect. “Hyperparameter Optimization”. It fine-tunes critical hyperparameters, like RNN layer units and learning rate, by iteratively refining a surrogate model to identify the optimal configuration minimizing the relative error metric. The specific parameters optimized include:

- Layer Units: The number of units in each RNN layer (LSTM, GRU, Bi-LSTM) to adjust the model’s complexity.
- Learning Rate: Adaptively tuned to enhance convergence and generalization.

#### Training process

Model training is conducted using the Adam optimizer (cf. Section “Hyperparameter Optimization”), with an adaptive learning rate, which facilitates stable convergence over the epochs. A key feature of this setup includes *LearnRateDropFactor* and *LearnRateDropPeriod* parameters dynamically adjust the learning rate, reducing it to approximately 36% of the initial value by the end of training. This prevents overfitting by allowing larger updates initially, then gradually refining the model.

#### Model evaluation and prediction

After training, the model undergoes evaluation using a distinct set of test points. The mean relative error (cf. (1)) is calculated to quantify the discrepancies between predicted and true (EM-evaluated) frequency responses. This metric, averaged across the four outputs, serves as an overall measure of model performance. Predictions, encompassing both real and imaginary components in complex format, are saved along with the trained models to design paths for future analysis and validation.

**Fig. 8:**
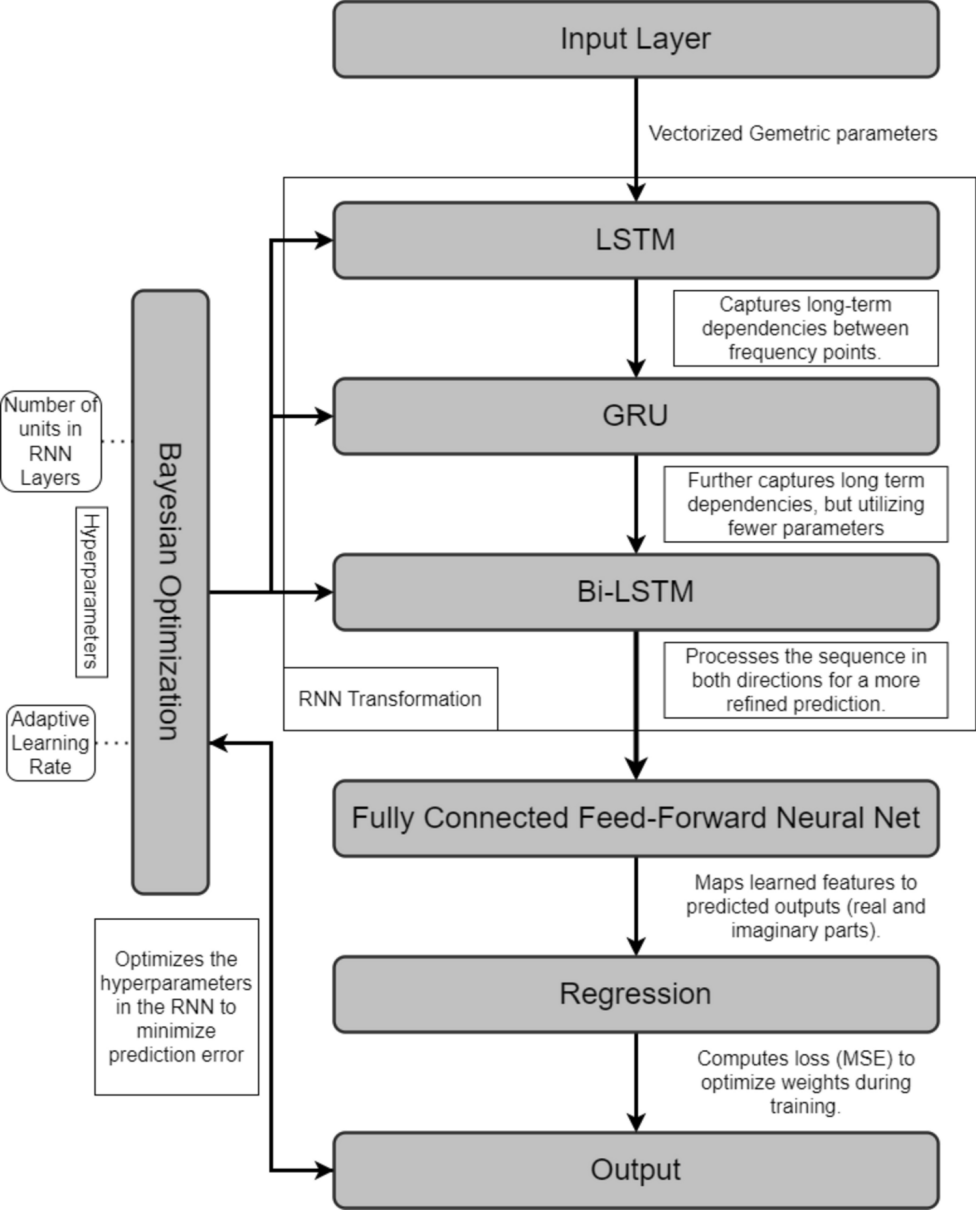
Data flow diagram of the proposed RNN surrogate model.

**Fig. 9:**
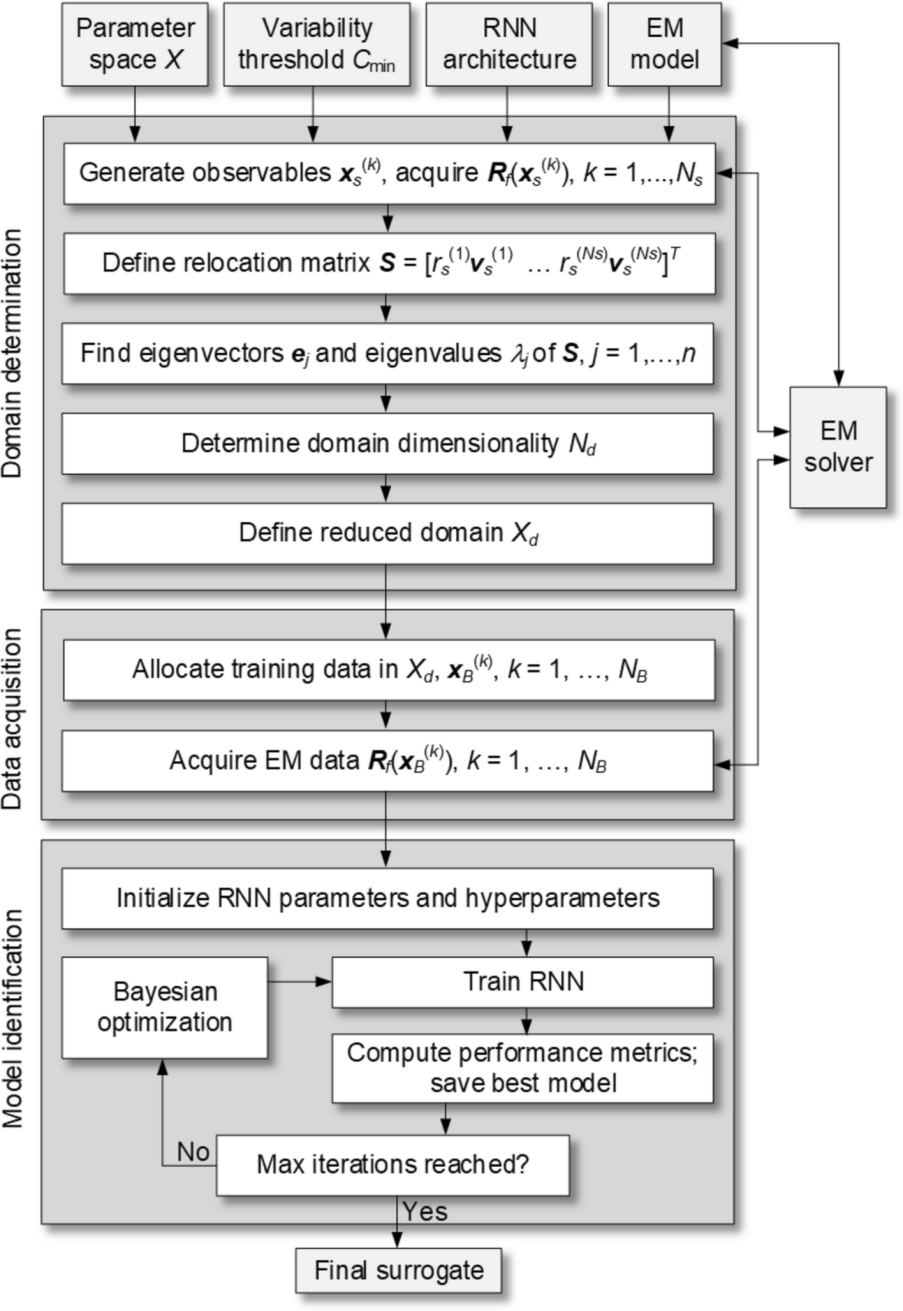
Flowchart of the overall modeling process using the approach suggested in this research.

For a supplementary clarification, Fig. 8 shows the diagram explaining the data flow in the proposed model. Furthermore, Fig. 9 illustrates the operating flow of the complete model construction process, which includes the establishment of the confined domain, sampling, acquisition of the EM simulation data, and identification of the RNN metamodel.

## Results

The proposed modeling methodology is demonstrated using three circuits. It is also juxtaposed against several benchmark procedures. The material is organized as follows. Section “Benchmark Methods” outlines the benchmark techniques. Section “Setup” covers the setup. The results and discussion are provided in Sects. “Results” and Sect. “Discussion”. Application of the model for circuit optimization and experimental validation of the optimized circuits are included in Sect. “Applications Case Studies and Experimental Validation”.

### Benchmark methods

The benchmark methods are outlined in Table 1. These include kriging interpolation, radial basis functions, Gaussian process regression, support vector machines, and artificial neural networks (ANN). The last three models are deep feedforward ANN architectures added to provide a more meaningful comparison with the proposed RNN-based model and to demonstrate that sequential processing of frequency data does provide distinctive benefits, which cannot be achieved by merely increasing the network architecture complexity. These frameworks are widely used in high-frequency engineering in diverse applications (general-purpose modeling, global and multi-objective optimization, and machine learning). Consequently, they can be considered representative in the considered field. The cited references^109–112^ provide more extensive information about each method.

**Table 1:** Benchmark methods: the outline.

Method	Setup
Kriging^109^	• Second-order polynomial as a trend function • Gaussian correlation function • Hyperparameters found through maximum likelihood optimization
Radial basis functions (RBF)^109^	• Gaussian basis functions • Scaling coefficient adjusted through cross-validation
Gaussian process regression (GPR)^110^	• Rational Quadratic kernel functions • Separate GPR models for real and imaginary parts of the response Hyperparameters optimized through maximum likelihood estimation
Support vector regression (SVR)^111^	• Gaussian (RBF) kernel function • Separate SVM models for real and imaginary parts of the response Kernel scale optimized automatically for each frequency
Artificial neural network (ANN 1)^112^	• Fully connected architecture with ReLU activation • Layers: 512 → 256 → 128 → 64 → output (real + imaginary) Trained using the Adam optimizer with 400 epochs
ANN 2^112^	• Fully connected architecture with ReLU activation • Layers: 128 → 128 → 128 → 128 → 128 → 128 → output (real + imaginary) • Trained using the Adam optimizer with 400 epochs
ANN 3 ^112^	• Fully connected architecture with ReLU activation • Layers: 256 → 256 → 256 → 256 → 256 → output (real + imaginary) • Trained using the Adam optimizer with 400 epochs

### Setup

The suggested technique is employed to build surrogate models for the circuits discussed in Sects. “Example I” through Example III. To comprehensively demonstrate the properties of our approach, the surrogates are rendered in both the conventional space *X* and the reduced domain *X*_*d*_. This enables observing the advantages of the RNN-based surrogate and the benefits of combining it with dimensionality reduction.

The dimensionality of *X*_*d*_ is determined using FGSA with *C*_min_ = 0.9 (Sect. “Domain Confinement Using Global Sensitivity Analysis”). The models are built using datasets of different sizes, from 50 to 800 samples. The training points are allocated using LHS ^107^. The model accuracy is evaluated using the RRMSE defined in Sect. “Microwave Modelling” based on 100 independent testing samples (see also (1)). The hyperparameter space has also been summarized in Table 2.

**Table 2:** Hyperparameter space of the proposed RNN-based surrogate model.

Hyperparameters	Range
LSTM Units	(500, 750)
GRU Units	(500, 1000)
Bi-LSTM Units	(500, 1000)
Learning Rate	(0.0005, 0.00075)

The specific model setup is as follows:

- **Architecture (**as described in Sect. “RNN-based Surrogates with Sequential Processing of Frequency Responses”**)**: A deep Recurrent Neural Network comprising LSTM, GRU, and Bi-LSTM layers, followed by a Fully Connected output layer and a Regression layer to predict complex-valued antenna frequency responses.
- **Hyperparameter Tuning**: Utilizes Bayesian Optimization to fine-tune the number of units in each layer and the learning rate. The number of BO iterations was set to 25.
- **Layer arrangement**: 500 − 750 (LSTM) → 500–1000 (GRU) → 500 − 1000 (Bi-LSTM) → output (real + imaginary).
- **Training Configuration**: The model is trained for up to 10,000 epochs using the Adam optimizer, with early stopping and learning rate scheduling to prevent overfitting.

Comparing the training time and computational complexity, in general, we can safely state that the first four benchmark models (kriging, RBF, GPR, SVR) are cheap to construct. These models rely on relatively simple optimization tasks, such as maximum likelihood estimation for kernel-based techniques, which typically complete within seconds to a few minutes, depending on dataset size their training times are negligible compared to the time required to acquire electromagnetic (EM) simulation data, which can take several minutes per simulation. In comparison, the ANN (the fifth benchmark method) training times range from 2–3 min (for 50-point training set) to approximately an hour for the largest datasets. However, it remains computationally efficient due to its fixed architecture and the use of backpropagation for optimization.

In contrast, the proposed model requires significantly longer training times due to Bayesian optimization, which involves multiple network training cycles for hyperparameter tuning. For the smallest dataset (50 samples), training takes approximately 40 min, while for the largest dataset (800 samples), the process extends to tens of hours. Training time also varies based on the complexity of the test case, particularly the input space dimensionality. For instance, training on an 800-sample dataset requires around 12 h for Antenna I (six design variables), approximately 24 h for Antenna II (eleven variables), and 16 h for Antenna III (seven variables). Despite the increased training time, this computational cost remains a fraction of the overall time required for data acquisition. Additionally, this trade-off is justified by the significant improvements in predictive accuracy and generalization achieved by the proposed model.

### Results

Here, we report the results obtained for the considered test circuits using the proposed modeling approach and the benchmark procedures. These results are analyzed in depth in Sect. “Discussion”. Design applications and experimental validation of the circuits optimized using our surrogates for selected target operating parameters are covered in Sect. “Applications Case Studies and Experimental Validation”. Note that the test cases are challenging from the modelling perspective primarily due to wide ranges of parameters and frequency, and considerable nonlinearity of circuit’s responses.

#### Example I

**Fig. 10:**
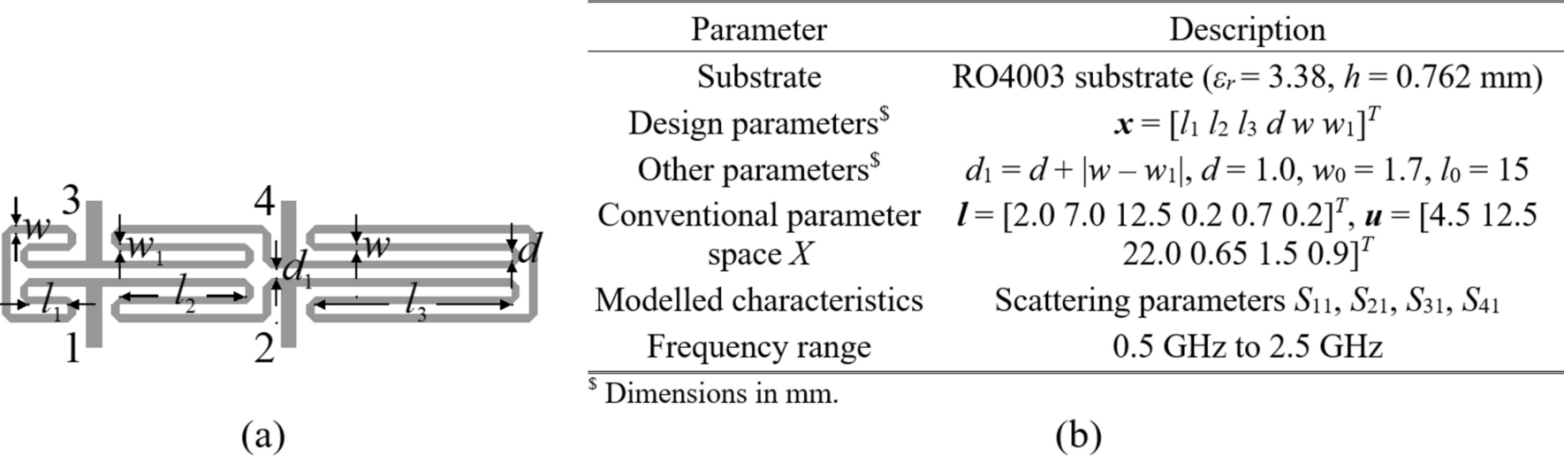
Compact coupler (Circuit I): (**a**) parameterized architecture, (**b**) parameters.

**Fig. 11:**
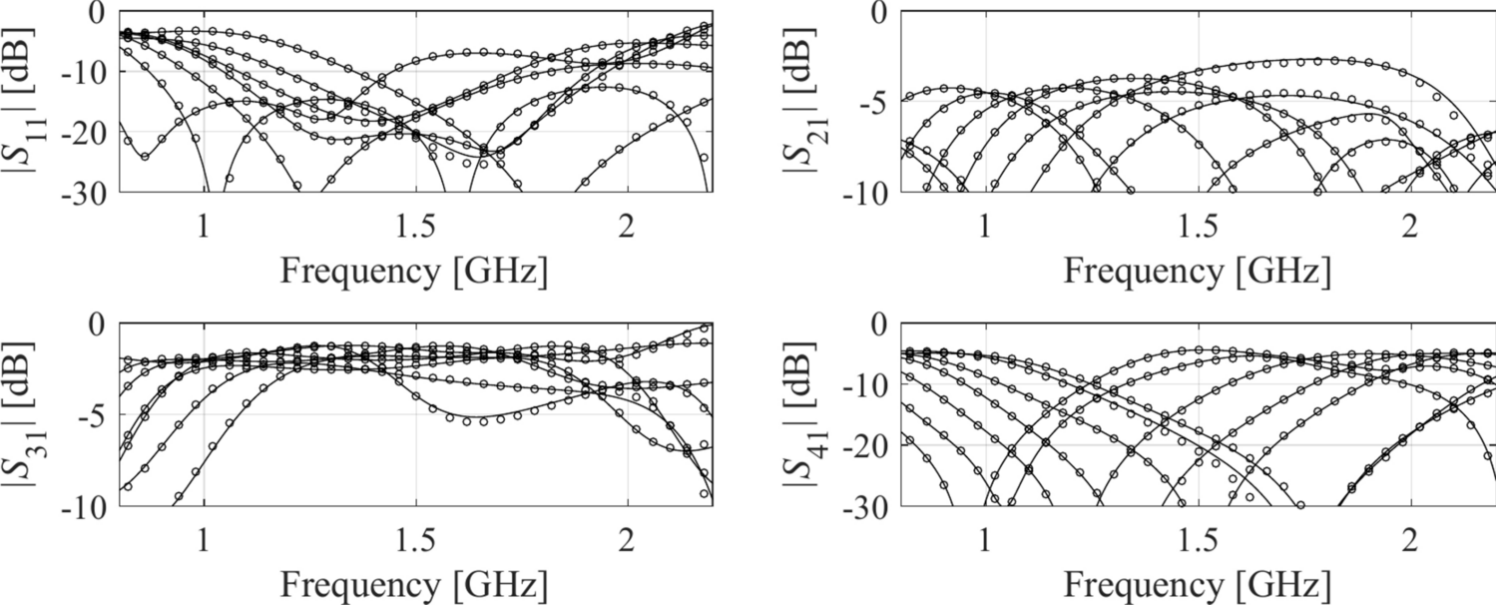
Circuit I: *S*-parameters versus frequency at selected test designs: surrogate-predicted (o) and EM-evaluated responses (—). The model was built using *N*_*B*_ = 800 samples.

The first verification case is a compact coupler with unequal power division ratio shown in Fig. 10^113^. The same figure also provides data on design variables, parameter bounds, and the target frequency range . The objective is to construct the model of scattering parameters *S*_11_, *S*_21_, *S*_31_, and *S*_41_. The sensitivity analysis has been carried out based on fifty random samples, yielding the normalized eigenvalues are *λ*_1_ = 1.00, *λ*_2_ = 0.65, *λ*_3_ = 0.51, *λ*_4_ = 0.46, *λ*_5_ = 0.37, *λ*_6_ = 0.28, which leads to the dimensionality of the reduced domain equal to *N*_*d*_ = 3, for which we have ${{\sqrt {\sum\nolimits_{j = 1}^{{N_{d} }} {\lambda_{j}^{2} } } } \mathord{\left/ {\vphantom {{\sqrt {\sum\nolimits_{j = 1}^{{N_{d} }} {\lambda_{j}^{2} } } } {\sqrt {\sum\nolimits_{j = 1}^{n} {\lambda_{j}^{2} } } }}} \right. \kern-0pt} {\sqrt {\sum\nolimits_{j = 1}^{n} {\lambda_{j}^{2} } } }} = 0.89$ (almost equal to the acceptance threshold *C*_min_ = 0.9). The results are encapsulated in Table 3. The surrogate-predicted and EM-simulated scattering parameters for the proposed model built in the reduced domain with *N*_*B*_ = 800 samples can be found in Fig. 11.

**Table 3:** Numerical results for Example I

Domain	Modeling method	Average relative RMS error				
		*N*_*B*_ = 50	*N*_*B*_ = 100	*N*_*B*_ = 200	*N*_*B*_ = 400	*N*_*B*_ = 800
Original (*X*)	Kriging	25.7%	17.9%	13.5%	9.9%	8.0%
	RBF	28.3%	19.1%	13.9%	10.3%	8.9%
	GPR	30.9%	21.6%	19.5%	15.5%	10.4%
	SVR	37.6%	26.2%	25.3%	21.2%	16.1%
	ANN 1	29.8%	10.5%	10.8%	7.6%	5.9%
	ANN 2	39.8%	32.3%	18.3%	12.5%	9.8%
	ANN 3	37.6%	31.8%	25.6%	13.8%	11.2%
	RNN-LSTM (this work)	24.2%	13.4%	9.3%	7.2%	5.3%
Reduced (*X*_*d*_)	Kriging	5.9%	3.8%	2.7%	2.4%	1.8%
	RBF	8.0%	5.7%	3.4%	3.0%	2.4%
	GPR	9.7%	7.2%	5.3%	4.1%	3.0%
	SVR	15.5%	10.3%	7.5%	5.4%	4.3%
	ANN 1	5.9%	4.1%	2.8%	2.4%	1.9%
	ANN 2	7.3%	8.3%	4.0%	3.4%	2.5%
	ANN 3	6.5%	5.8%	4.8%	3.3%	2.1%
	RNN-LSTM (this work)	5.5%	3.1%	2.8%	2.2%	1.9%

#### Example II

**Fig. 12:**
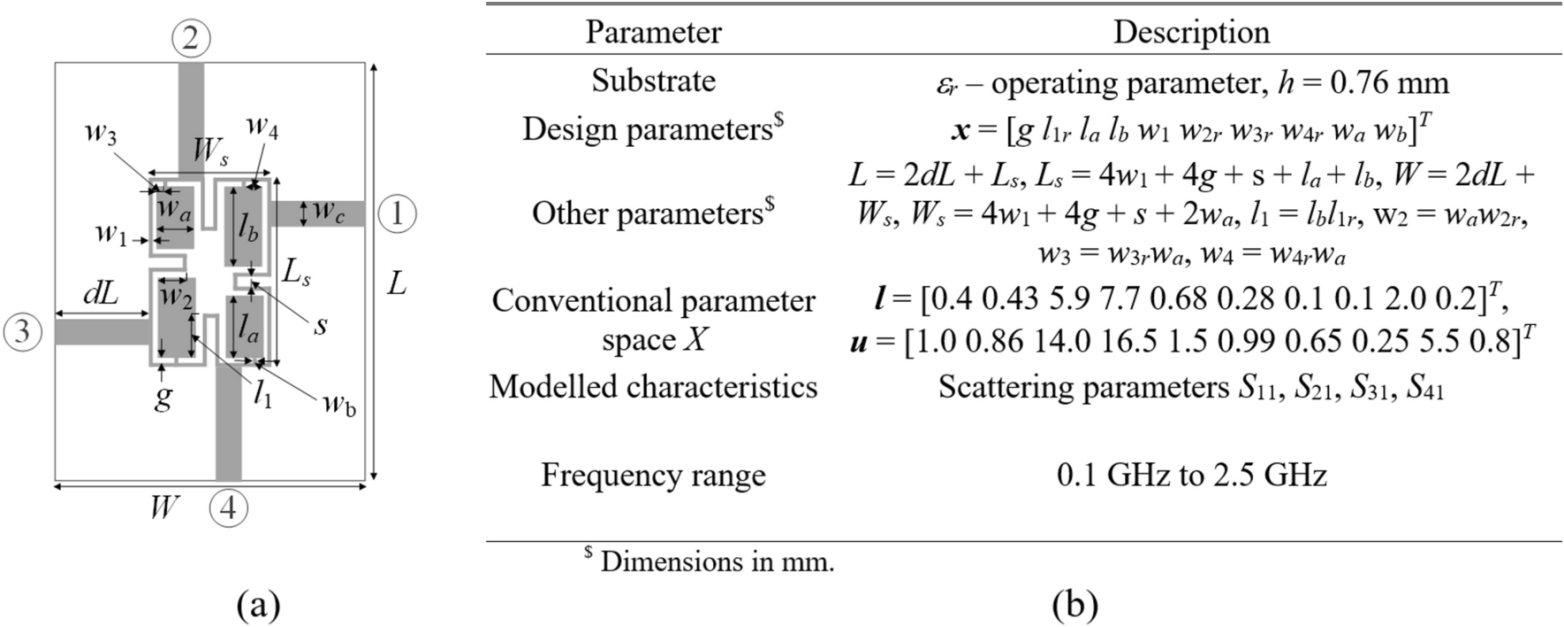
Compact branch-line coupler (Circuit II): (**a**) parameterized architecture, (**b**) parameters.

The second test case is a compact branch-line coupler with compact microstrip resonant cells (CMRCs)^114^ shown in Fig. 12. As for Example I, we aim to build the surrogate that represents scattering parameters *S*_11_, *S*_21_, *S*_31_, and *S*_41_. The substrate’s permittivity is an extra design parameter considered in the range from 2.0 to 5.0 so that the model covers diverse substrate materials.

**Fig. 13:**
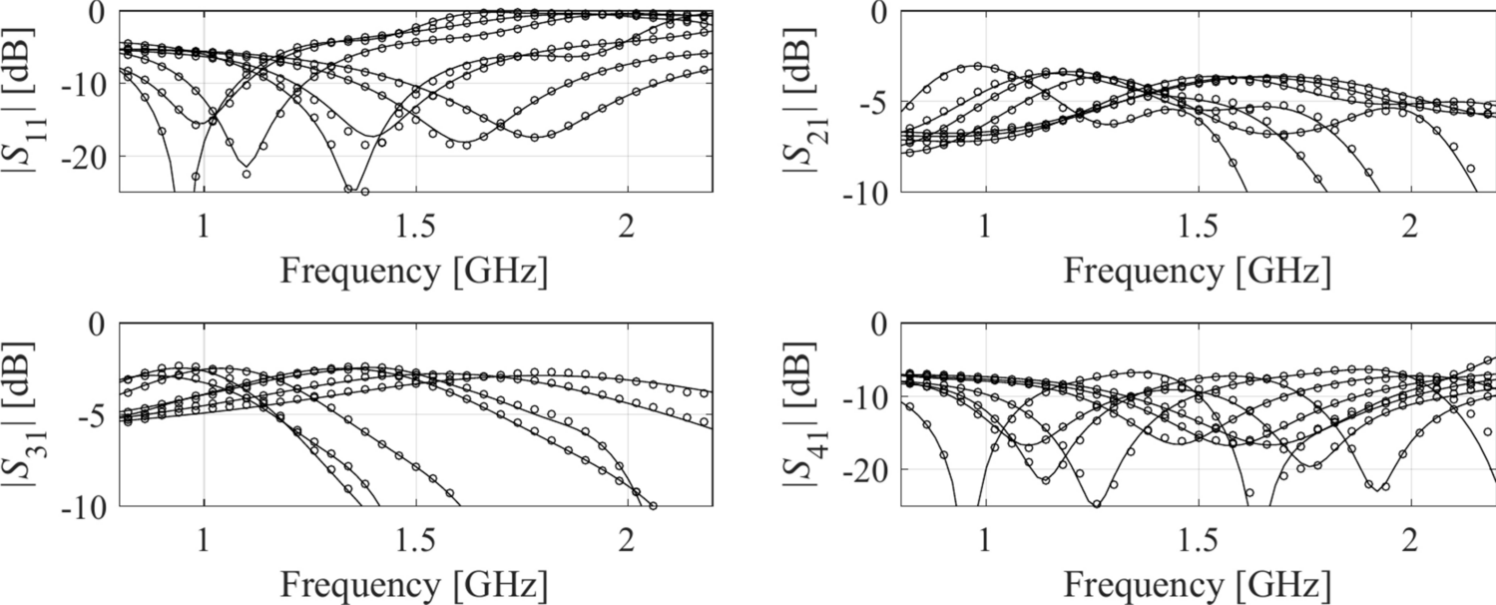
Circuit II: *S*-parameters versus frequency at selected test designs: surrogate-predicted (o) and EM-simulated responses (—). The surrogate was constructed with *N*_*B*_ = 800 training samples.

FGSA run based on fifty random samples, yields the normalized eigenvalues *λ*_1_ = 1.00, *λ*_2_ = 0.66, *λ*_3_ = 0.54, *λ*_4_ = 0.48, *λ*_5_ = 0.41, *λ*_6_ = 0.39, *λ*_7_ = 0.30,*λ*_8_ = 0.25,*λ*_9_ = 0.22,*λ*_10_ = 0.16,*λ*_11_ = 0.13. The resulting domain dimensionality is *N*_*d*_ = 4 with ${{\sqrt {\sum\nolimits_{j = 1}^{{N_{d} }} {\lambda_{j}^{2} } } } \mathord{\left/ {\vphantom {{\sqrt {\sum\nolimits_{j = 1}^{{N_{d} }} {\lambda_{j}^{2} } } } {\sqrt {\sum\nolimits_{j = 1}^{n} {\lambda_{j}^{2} } } }}} \right. \kern-0pt} {\sqrt {\sum\nolimits_{j = 1}^{n} {\lambda_{j}^{2} } } }} = 0.88$. The results can be found in Table 4, whereas Fig. 13 shows surrogate-predicted and EM-simulated *S*-parameters for the metamodel rendered using *N*_*B*_ = 800 training.

**Table 4:** Numerical results for test Example II.

Domain	Modeling method	Average relative RMS error				
		*N*_*B*_ = 50	*N*_*B*_ = 100	*N*_*B*_ = 200	*N*_*B*_ = 400	*N*_*B*_ = 800
Original (*X*)	Kriging	52.3%	38.3%	31.0%	27.3%	23.3%
	RBF	51.8%	40.5%	37.4%	32.8%	27.2%
	GPR	45.6%	40.6%	35.2%	31.5%	27.2%
	SVR	50.1%	45.0%	39.6%	35.9%	31.6%
	ANN 1	63.9%	45.3%	27.3%	15.0%	9.0%
	ANN 2	60.4%	48.3%	35.2%	18.3%	13.9%
	ANN 3	64.8%	46.3%	32.3%	17.9%	14.5%
	RNN-LSTM (this work)	45.3%	31.9%	19.4%	10.7%	8.4%
Reduced (*X*_*d*_)	Kriging	21.5%	15.8%	11.1%	8.5%	6.4%
	RBF	23.2%	17.0%	13.0%	9.8%	7.3%
	GPR	33.5%	21.5%	15.8%	11.2%	8.6%
	SVR	36.0%	29.4%	23.8%	19.3%	16.1%
	ANN 1	13.9%	9.7%	6.7%	4.8%	4.1%
	ANN 2	12.7%	11.6%	7.3%	5.4%	4.6%
	ANN 3	11.3%	10.8%	8.3%	6.2%	4.1%
	RNN-LSTM (this work)	14.0%	7.9%	5.4%	4.2%	3.9%

#### Example III

**Fig. 14:**
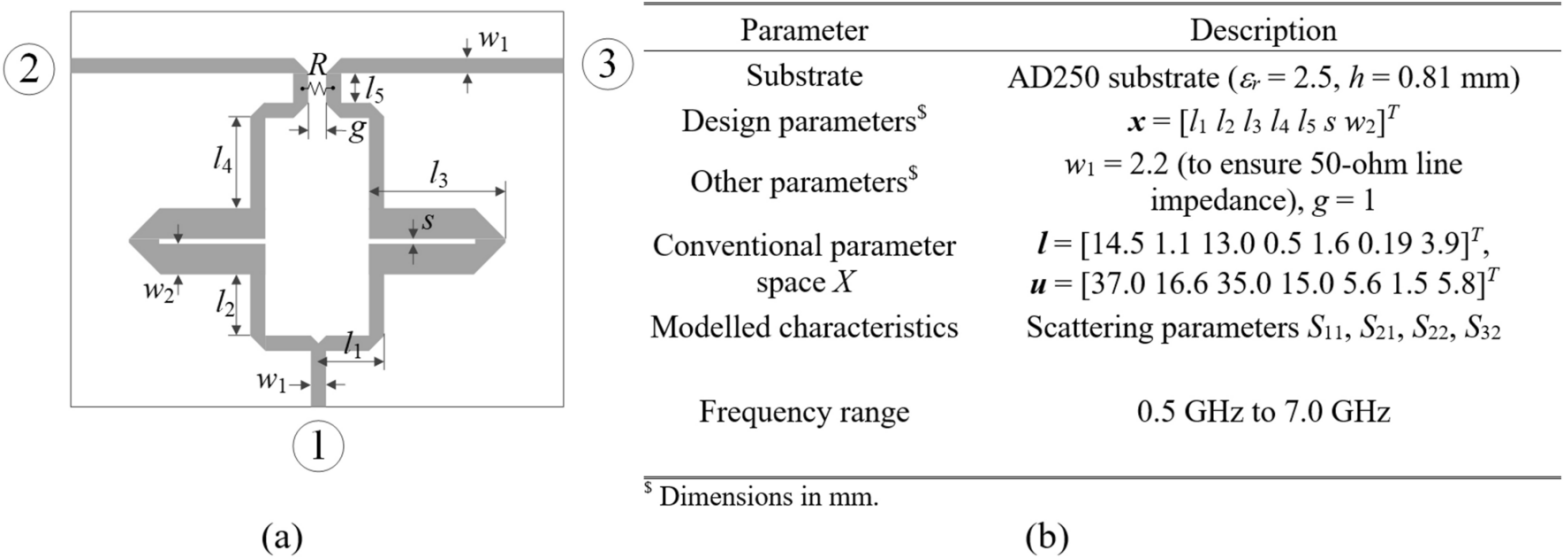
Dual-band power divider (Circuit III): (**a**) parameterized architecture, (**b**) parameters.

**Fig. 15:**
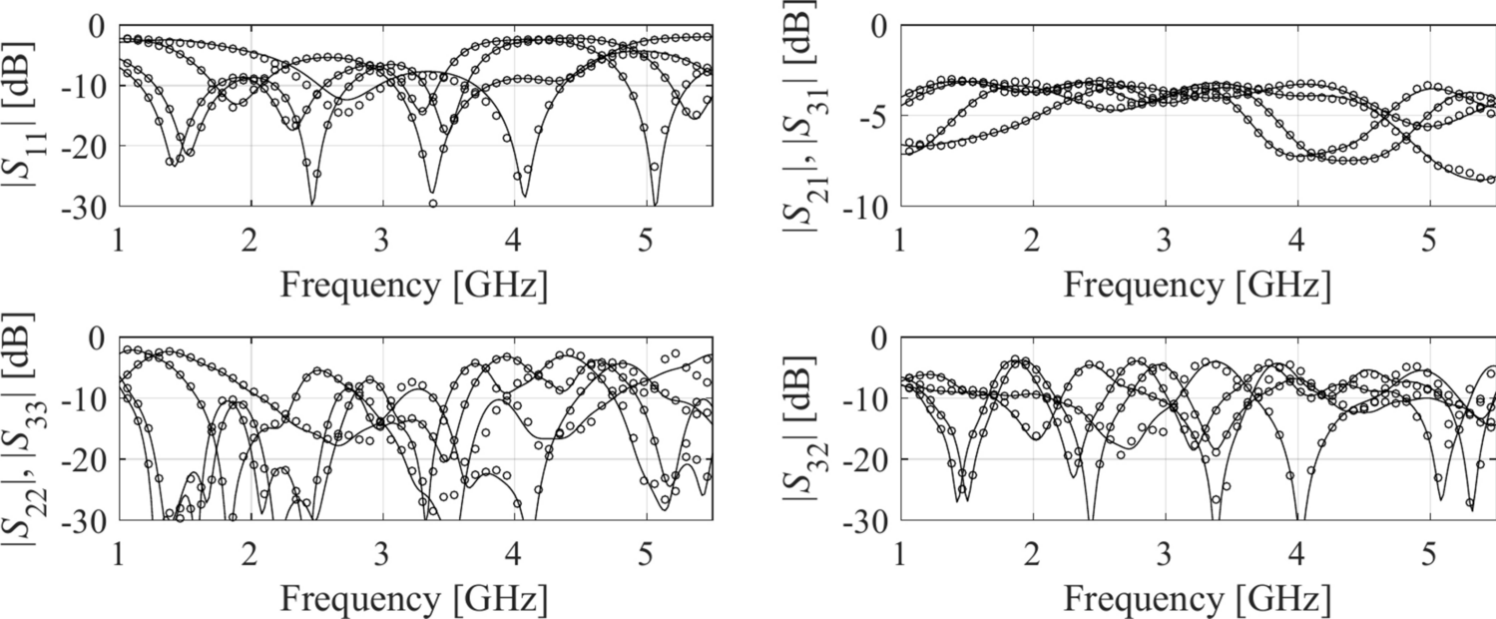
Circuit III: *S*-parameters versus frequency at selected test designs: surrogate-predicted (o) and EM-simulated responses (—). The surrogate was constructed with *N*_*B*_ = 800 training samples.

The last test example is a dual-band power divider illustrated in Fig. 14^115^. For this circuit, the goal is to build the metamodel representing the *S*-parameters: *S*_11_, *S*_21_, *S*_22_, and *S*_32_. Just as for other examples, FGSA was run using fifty random samples. The normalized eigenvalues are *λ*_1_ = 1.00, *λ*_2_ = 0.77, *λ*_3_ = 0.66, *λ*_4_ = 0.64, *λ*_5_ = 0.50, *λ*_6_ = 0.48, *λ*_7_ = 0.45. The reduced domain dimensionality is *N*_*d*_ = 4, which gives ${{\sqrt {\sum\nolimits_{j = 1}^{{N_{d} }} {\lambda_{j}^{2} } } } \mathord{\left/ {\vphantom {{\sqrt {\sum\nolimits_{j = 1}^{{N_{d} }} {\lambda_{j}^{2} } } } {\sqrt {\sum\nolimits_{j = 1}^{n} {\lambda_{j}^{2} } } }}} \right. \kern-0pt} {\sqrt {\sum\nolimits_{j = 1}^{n} {\lambda_{j}^{2} } } }} = 0.89$. The numerical results are provided in Table 5. Note that this is the most challenging example, therefore, an extended training set of 1600 samples was considered as well. The circuit responses at the selected test design are showcased in Fig. 15.

**Table 5:** Numerical results for test Case III.

Domain	Modeling method	Average relative RMS error					
		*N*_*B*_ = 50	*N*_*B*_ = 100	*N*_*B*_ = 200	*N*_*B*_ = 400	*N*_*B*_ = 800	*N*_*B*_ = 1600
Original (*X*)	Kriging	63.6%	53.8%	45.2%	40.0%	35.1%	32.3%
	RBF	68.9%	55.2%	43.9%	40.8%	37.2%	34.7%
	GPR	70.2%	69.1%	56.4%	47.9%	42.3%	70.2%
	SVR	79.6%	72.6%	68.4%	63.2%	61.0%	59.6%
	ANN 1	78.3%	77.4%	65.2%	59.4%	37.2%	33.3%
	ANN 2	82.2%	78.3%	66.2%	61.2%	49.8%	42.3%
	ANN 3	79.3%	77.8%	64.8%	59.7%	45.5%	44.1%
	RNN-LSTM (this work)	75.3%	55.6%	37.8%	26.8%	17.8%	16.3%
Reduced (*X*_*d*_)	Kriging	38.9%	28.7%	23.5%	16.6%	12.5%	8.4%
	RBF	42.5%	31.3%	26.0%	18.1%	14.1%	9.9%
	GPR	60.7%	51.8%	41.1%	41.1%	33.7%	25.8%
	SVR	55.5%	51.9%	47.7%	44.0%	39.1%	37.2%
	ANN 1	52.4%	34.0%	23.2%	16.8%	12.0%	10.0%
	ANN 2	47.5%	35.7%	24.6%	19.5%	14.6%	11.1%
	ANN 3	48.7%	36.2%	25.1%	18.7%	15.1%	11.4%
	RNN-LSTM (this work)	50.7%	29.3%	18.8%	11.9%	7.6%	5.6%

### Discussion

The numerical data reported in Tables 3, 4, and 5 unequivocally demonstrates the competitive operation of the presented modeling methodology compared to the benchmark. For all verification structures, the RNN-based surrogate offers improved predictive power, with the advantage over comparison methods being quite significant for certain combinations of circuits and the training dataset sizes. The benefits of dimensionality reduction are also evident and contribute to dramatically improving accuracy, with RRMSE being lower than two percent for Circuit I and slightly above five percent for the most extensive training sets. This level of reliability makes the surrogates suitable for design purposes, which will be discussed in Sect. “Applications Case Studies and Experimental Validation”. The accuracy improvements are noticeable for the lowest-cardinality training sets (50 and 100 samples) but even more pronounced for *N*_*B*_ = 400 and 800. This underscores the relevance of sequential processing of frequency characteristics leveraged by our methodology and realized using a dedicated combination of LSTM and GRU layers. It should also be noted that apart from Circuit I (the most straightforward test case), modeling in the conventional parameter space is not feasible, i.e., the values of RRMSE exceed twenty (Circuit II) and thirty percent (Circuit III), even for *N*_*B*_ ≥ 800. It can also be observed that deep feedforward ANN models (ANN 1, 2, and 3) do not perform as well as the proposed RNN-based technique, which is indicative of the fact that just increasing the network complexity does not carry over to competitive results, such as those obtained using the proposed methodology. It appears that sequential treatment of the frequency data does have a positive impact on the results.

Another appealing feature of our framework is consistency of results. Our method has been shown superior over all benchmark techniques, for all test circuits, training set sizes, and the selection of the domain (original or reduced). In addition, it exhibits excellent scalability, i.e., fast enhancement of the predictive power as a function of the training dataset cardinality. This is particularly noticeable for a dimensionality-reduced domain. Therein, the relationship between the mean distance between the training sites scales more favorably with *N*_*B*_. Again, the mentioned benefits can be attributed to the specific architecture of the proposed RNN-based surrogate and the advantages of sequential processing of circuit response data (as a function of frequency). Our approach differs from standard modeling approaches (kernel-based and neural-network-based regression techniques), where the circuit characteristics are treated as vector-valued ensembles with frequency often considered a supplementary parameter.

### Applications case studies and experimental validation

The surrogate models generated using the proposed methodology were employed to perform parameter tuning of Circuits I, II, and III. The exemplary design scenarios assumed for all circuits are detailed in Table 6. The table showcases the optimized geometry variable vectors obtained by directly optimizing the respective metamodels (with no further correction). The surrogated constructed with *N*_*B*_ = 800 training samples were employed in all cases. To provide additional illustration, the optimized designs were fabricated and experimentally validated.

**Table 6:** Optimization of Circuits I, II, and III using the suggested surrogate model.

Circuit	Target frequencies	Optimization goals	Target substrate	Optimum design
I	*f*_0_ = 1.0 GHz	Improve |*S*_11_| and |*S*_41_| at *f*_0_; maintain 3 dB power split ratio (|*S*_21_| – |*S*_31_|= 3 dB) at *f*_0_	RO4003 (*ε*_*r*_ = 3.38, *h* = 0.76 mm)	x* = [2.1 10.3 20.0 0.5 1.35 0.6]^*T*^
II	*f*_0_ = 1.25 GHz	Improve |*S*_11_| and |*S*_41_| at *f*_0_; maintain equal power split ratio (|*S*_21_| =|*S*_31_|) at *f*_0_	RO4003 (*ε*_*r*_ = 3.38, *h* = 0.76 mm)	x* = [0.4 0.65 12.5 13.5 0.9 0.4 0.25 0.16 3.31 0.55]^*T*^
III	*f*_1_ = 1.5 GHz *f*_2_ = 2.45 GHz	Improve matching |*S*_11_|, |*S*_22_| =|*S*_33_|, and port isolation |*S*_21_| =|*S*_32_| at both *f*_1_ and *f*_2_; maintain equal power division ratio |*S*_21_| =|*S*_31_| at *f*_1_ and *f*_2_	AD250 (*ε*_*r*_ = 2.5, *h* = 0.81 mm)	x* = [31.3 9.8 31.2 8.85 4.6 0.7 4.3]^*T*^

**Fig. 16:**
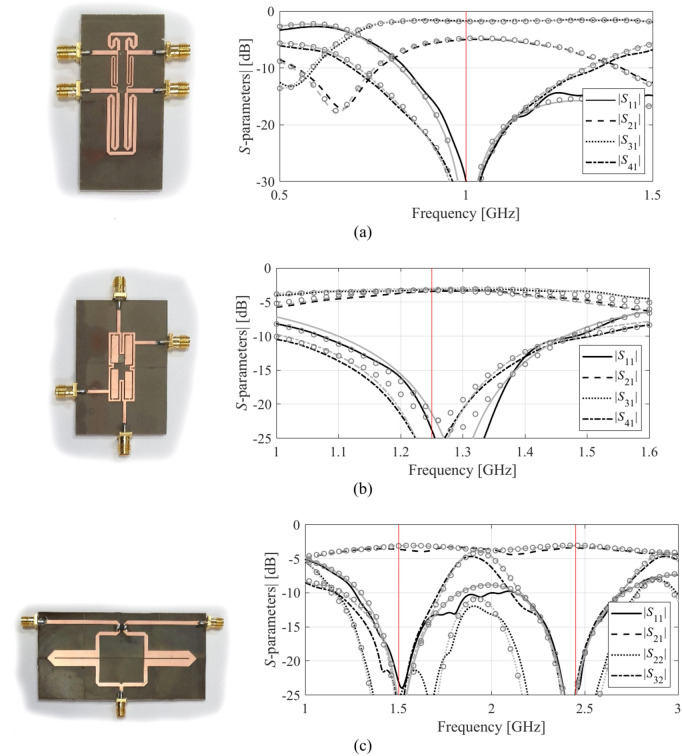
Application case studies (circuit optimization). The pictures show the circuit prototypes corresponding to metamodel-optimized designs gathered in Table 6 and their frequency characteristics: surrogate prediction (o), EM simulation (grey), and measurement (black): (**a**) Circuit I, (**b**) Circuit II, (**c**) Circuit III.

The photographs of the circuit prototypes and their *S*-parameter responses, as predicted by the surrogate, by EM simulation, and evaluated through measurements, are shown in Fig. 16. Observe a good agreement between the model predictions and EM analysis. The alignment between EM simulations and experimental results is also satisfactory. Minor discrepancies are due to manufacturing inaccuracies and the effects of the SMA connectors.

## Conclusion

This study focused on developing an improved methodology for reliable modeling of passive microwave circuits. The approach suggested here leverages the properties of recurrent neural networks (RNNs) employed to process information enclosed in the circuit’s electrical characteristics efficiently. Our technique’s distinctive feature is handling frequency as a sequential parameter, which facilitates building a behavioral (data-driven) system representation. This contrasts with more conventional methods, where frequency responses are treated in parallel (as vector-valued entities), with the frequency often treated as an independent parameter. The proposed RNN architecture incorporates LSTM and GRU layers and the bidirectional LSTM layer to capture frequency-wise dependencies within the system’s outputs. The major hyperparameters of the network are adjusted using Bayesian Optimization (BO). Flexible dimensionality reduction is another tool employed to enhance the model’s quality significantly. It is realized using rapid global sensitivity analysis and implemented to allow arbitrary orientation of the reduced domain (spanned by the vectors associated with the maximum circuit response variations) but without eliminating individual design variables.

Our methodology has been extensively verified using several planar circuits and a range of benchmark methods. To ensure meaningful assessment, the surrogate models were constructed in conventional and dimensionality-reduced domains using training sets of sizes from 50 to 800 samples. The results unanimously demonstrate the advantages of our procedure, which turned out to be superior to all benchmark techniques regarding the surrogate’s predictive power measured using the relative root mean square error (RRMSE). These benefits are consistent for all considered test circuits, domain selection, training dataset cardinality, and the scalability of the model’s accuracy concerning the number of training samples. They also corroborate the adequacy of the assumed RNN architecture and the introduced data handling paradigm. Future work will be oriented toward further improvements of the procedure’s reliability and extending its applicability to higher-dimensional problems.

## Data Availability

The datasets used and/or analyzed during the current study are available from the corresponding author on reasonable request.

## References

[1] ADS (Advanced Design System) Keysight Technologies Fountaingrove Parkway 1400 Santa Rosa CA 95403–1799 2023.

[2] AWR Microwave Office Cadence Design Systems Inc. San Jose, CA 95134 USA 2023

[3] CST Microwave Studio, https://www.3ds.com/products-services/simulia/products/cst-studio-suite/, Dassault Systems Rue Marcel Dassault 78140 Velizy-Villacoublay, France 2023.

[4] HFSS, ANSYS, http://www.ansoft.com/products/hf/hfss/, 2600 Ansys Dr., Canonsburg PA 15317 USA 2023.

[5] Xu, X., Yuan, T., Wu, J., Liu, J. & Du, Y. Detection of trace dibutyl phthalate based on multiresonant terahertz metamaterial sensor. *IEEE Sens. J.* **24**(2), 1415–1423 (2024).

[6] Herraiz, D., Esteban, H., Herraiz, D., Belenguer, A. & Boria, V. E. A novel in-line ESICL-to-ESIW transition for high-performance empty substrate integrated microwave systems. *IEEE Microw. Wirel. Techn. Lett.* **34**(4), 363–366 (2024).

[7] Tan, X. & Zhang, Y. A Compact rat-race coupler with widely tunable frequency- and power-dividing ratio. *IEEE Microw. Wirel. Techn. Lett.* **34**(1), 17–20 (2024).

[8] Wu, D.-S., Li, Y. C., Xue, Q. & Mou, J. “LTCC bandstop filters with controllable bandwidths using transmission zeros pair”, *IEEE Trans*. *Circuits Syst. II: Express Briefs* **67**(6), 1034–1038 (2020).

[9] Sen, S. & Moyra, T. “Compact microstrip low-pass filtering power divider with wide harmonic suppression”, *IET Microwaves*. *Ant. Propag.* **13**(12), 2026–2031 (2019).

[10] Dong, Y., Yang, B., Yu, Z. & Zhou, J. Robust fast electromagnetic optimization of SIW filters using model-based deviation estimation and Jacobian matrix update. *IEEE Access* **8**, 2708–2722 (2020).

[11] Koziel, S., Pietrenko-Dabrowska, A. & Plotka, P. Design specification management with automated decision-making for reliable optimization of miniaturized microwave components. *Sc. Rep.* **12**, 829 (2022). 35039575 10.1038/s41598-022-04810-1 PMC8763926

[12] Ren, Z., He, S., Zhang, D., Zhang, Y. & Koh, C. S. A possibility-based robust optimal design algorithm in preliminary design state of electromagnetic devices. *IEEE Trans. Magn.* **52**(3), 7001504 (2016).

[13] Zhang, Z. et al. A surrogate modeling space definition method for efficient filter yield optimization. *IEEE Microw. Wirel. Techn. Lett.* **33**(6), 631–634 (2023).

[14] Cilici, F. et al. Nonintrusive machine learning-based yield recovery and performance recentering for mm-wave power amplifiers: a two-stage class-A power amplifier case study. *IEEE Trans. Microw. Theory Techn.* **72**(5), 3046–3064 (2024).

[15] Liu, S., Pei, C., Khan, L., Wang, H. & Tao, S. Multiobjective optimization of coding metamaterial for low-profile and broadband microwave absorber. *IEEE Ant. Wirel. Propag. Lett.* **23**(1), 379–383 (2024).

[16] Torun, H. M. & Swaminathan, M. High-dimensional global optimization method for high-frequency electronic design. *IEEE Trans. Microw. Theory Techn.* **67**(6), 2128–2142 (2019).

[17] Jiao, D. & V. C. do Nascimento,. Fast rank-revealing method for solving large global optimization problems and its applications. *IEEE Trans. Microw. Theory Techn.* **72**(8), 4555–4567 (2024).

[18] Koziel, S. & Pietrenko-Dabrowska, A. Rapid surrogate-aided multi-criterial optimization of compact microwave passives employing machine learning and ANNs. *IEEE Trans. Microw. Theory Techn.* **72**(8), 4475–4488 (2024).

[19] Luo, X., Yang, B. & Qian, H. J. Adaptive synthesis for resonator-coupled filters based on particle swarm optimization. *IEEE Trans. Microw. Theory Techn.* **67**(2), 712–725 (2019).

[20] Oyelade, O. N., Ezugwu, A.E.-S., Mohamed, T. I. A. & Abualigah, L. Ebola optimization search algorithm: a new nature-inspired metaheuristic optimization algorithm. *IEEE Access* **10**, 16150–16177 (2022).

[21] Li, X. & Luk, K. M. The grey wolf optimizer and its applications in electromagnetics. *IEEE Trans. Ant. Prop.* **68**(3), 2186–2197 (2020).

[22] Li, Y. & Luo, X. Adaptive synthesis using hybrid genetic algorithm and particle swarm optimization for reflectionless filter with lumped elements. *IEEE Trans. Microw. Theory & Techn.* **71**(12), 5317–5334 (2023).

[23] Xue, L. et al. An unsupervised microwave filter design optimization method based on a hybrid surrogate model-assisted evolutionary algorithm. *IEEE Trans. Microw. Theory Techn.* **71**(3), 1159–1170 (2023).

[24] Wang, J., Yang, X. S. & Wang, B. Z. Efficient gradient-based optimisation of pixel antenna with large-scale connections. *IET Microw. Ant. Prop.* **12**(3), 385–389 (2018).

[25] Koziel, S. & Pietrenko-Dabrowska, A. Variable-fidelity simulation models and sparse gradient updates for cost-efficient optimization of compact antenna input characteristics. *Sensors* **19**, 8 (2019). 10.3390/s19081806 PMC6515375 30991769

[26] Pietrenko-Dabrowska, A. & Koziel, S. Computationally-efficient design optimization of antennas by accelerated gradient search with sensitivity and design change monitoring. *IET Microw. Ant. Prop.* **14**(2), 165–170 (2020).

[27] Zhang, W. et al. EM-centric multiphysics optimization of microwave components using parallel computational approach. *IEEE Trans. Microw. Theory Techn.* **68**(2), 479–489 (2020).

[28] Feng, F. et al. Coarse- and fine-mesh space mapping for EM optimization incorporating mesh deformation. *IEEE Microw. Wireless Comp. Lett.* **29**(8), 510–512 (2019).

[29] Pietrenko-Dabrowska, A. & Koziel, S. *Response feature technology for high-frequency electronics Optimization modeling and design automation* (Springer, 2023).

[30] Zhang, C., Feng, F., Gongal-Reddy, V., Zhang, Q. J. & Bandler, J. W. Cognition-driven formulation of space mapping for equal-ripple optimization of microwave filters. *IEEE Trans. Microw. Theory Techn.* **63**(7), 2154–2165 (2015).

[31] Bandler, J. W. & Rayas-Sánchez, J. E. An early history of optimization technology for automated design of microwave circuits. *IEEE J. Microw.* **3**(1), 319–337 (2023).

[32] Wang, L. L., Yang, X. S. & Ma, C. J. An efficient gradient-based hybrid parameter-topology optimization for antenna design. *IEEE Trans. Ant. Propag.* **71**(12), 9477–9486 (2023).

[33] Li, J., Yang, A., Tian, C., Ye, L. & Chen, B. Multi-fidelity Bayesian algorithm for antenna optimization. *J Syst. Eng. Electr.* **33**(6), 1119–1126 (2022).

[34] Koziel, S. & Leifsson, L. *Simulation-driven design by knowledge-based response correction techniques* (Springer, 2016).

[35] Pietrenko-Dabrowska, A. & Koziel, S. Reliable surrogate modeling of antenna input characteristics by means of domain confinement and principal components. *Electronics* **9**(5), 1–16 (2020).

[36] Chellappa, S., Feng, L., de la Rubia, V. & Benner, P. Inf-sup-constant-free state error estimator for model order reduction of parametric systems in electromagnetics. *IEEE Trans. Microw. Theory Techn.* **71**(11), 4762–4777 (2023).

[37] Zhang, J., Feng, F. & Zhang, Q.-J. Rapid yield estimation of microwave passive components using model-order reduction based neuro-transfer function models. *IEEE Microw. Wirel. Comp. Lett.* **31**(4), 333–336 (2021).

[38] Wu, Y., Pan, G., Lu, D. & Yu, M. Artificial neural network for dimensionality reduction and its application to microwave filters inverse modeling. *IEEE Trans. Microw. Theory Techn.* **70**(11), 4683–4693 (2022).

[39] Chen, W. et al. Knowledge-guided and machine-learning-assisted synthesis for series-fed microstrip antenna arrays using base element modeling. *IEEE Trans. Ant. Propag.* **72**(2), 1497–1509 (2024).

[40] Babale, S. A. et al. Machine learning-based optimized 3G/LTE/5G planar wideband antenna with tri-bands filtering notches. *IEEE Access* **12**, 80669–80686 (2024).

[41] Feng, F. et al. Adaptive feature zero assisted surrogate-based EM optimization for microwave filter design. *IEEE Microw. Wirel. Comp. Lett.* **29**(1), 2–4 (2019).

[42] Mahmood, M., Koc, A., Morawski, R. & Le-Ngoc, T. Achieving capacity gains in practical full-duplex massive MIMO systems: a multi-objective optimization approach using hybrid beamforming. *IEEE Open J. Comm. Soc.* **5**, 2268–2286 (2024).

[43] Zhang, Z., Chen, H. C. & Cheng, Q. S. Surrogate-assisted quasi-Newton enhanced global optimization of antennas based on a heuristic hypersphere sampling. *IEEE Trans. Ant. Propag.* **69**(5), 2993–2998 (2021).

[44] Wu, Q., Wang, H. & Hong, W. Multistage collaborative machine learning and its application to antenna modeling and optimization. *IEEE Trans. Ant. Propag.* **68**(5), 3397–3409 (2020).

[45] Bilson, S., Hong Loh, T., Héliot, F. & Thompson, A. Physics-informed machine learning modelling of RF-EMF exposure in massive MIMO systems. *IEEE Access* **12**, 69410–69422 (2024).

[46] Wu, Q., Chen, W., Yu, C., Wang, H. & Hong, W. Multilayer machine learning-assisted optimization-based robust design and its applications to antennas and array. *IEEE Trans. Ant. & Propag.* **69**(9), 6052–6057 (2021).

[47] Pietrenko-Dabrowska, A., Koziel, S. & Golunski, L. Cost-efficient globalized parameter optimization of microwave components through response-feature surrogates and nature-inspired metaheuristics. *IEEE Access* **12**, 79051–79065 (2024).

[48] Manfredi, P. Probabilistic uncertainty quantification of microwave circuits using Gaussian processes. *IEEE Trans. Microw. Theory Techn.* **71**(6), 2360–2372 (2023).

[49] Feng, L., Benner, P., Romano, D. & Antonini, G. Matrix-free transfer function prediction using model reduction and machine learning. *IEEE Trans. Microw. Theory Techn.* **70**(12), 5392–5404 (2022).

[50] Cai, J., King, J., Yu, C., Liu, J. & Sun, L. Support vector regression-based behavioral modeling technique for RF power transistors. *IEEE Microw. & Wirel. Comp. Lett.* **28**(5), 428–430 (2018).

[51] Dong, J., Qin, W. & Wang, M. “Fast multi-objective optimization of multi-parameter antenna structures based on improved BPNN surrogate model. *IEEE Access* **7**, 77692–77701 (2019).

[52] Calik, N., Belen, M., Mahouti, P. & Koziel, S. Accurate modeling of frequency selective surfaces using fully-connected regression model with automated architecture determination and parameter selection based on Bayesian optimization. *IEEE Access* **9**, 38396–38410 (2021).

[53] Koziel, S., Mahouti, P., Calik, N., Belen, M. A. & Szczepanski, S. Improved modeling of miniaturized microwave structures using performance-driven fully-connected regression surrogate. *IEEE Access* **9**, 71470–71481 (2021).

[54] Jia, Q., Li, J., Wei, C. & Liu, J. Microwave photonic reconfigurable high precision instantaneous frequency measurement system assisted by stacking ensemble learning method. *J. Lightwave Techn.* **41**(6), 1696–1703 (2023).

[55] Chen, W., Ji, Y., Qi, Z., Yan, L. & Zhao, X. Automatic determination of set of multivariate basis polynomials based on recursion and application of LSPCR to high-dimensional uncertainty quantification of multi-conductor transmission lines. *IEEE Access* **12**, 18536–18544 (2024).

[56] Pang, Y., Zhou, B. & Nie, F. “Simultaneously learning neighborship and projection matrix for supervised dimensionality reduction”. *IEEE Trans Neural Netw. & Learn. Syst.* **30**(9), 2779–2793 (2019). 10.1109/TNNLS.2018.2886317 30640633

[57] Lv, Z., Wang, L., Han, Z., Zhao, J. & Wang, W. Surrogate-assisted particle swarm optimization algorithm with Pareto active learning for expensive multi-objective optimization. *IEEE/CAA J. Automatica Sinica* **6**(3), 838–849 (2019).

[58] Müller, D., Soto-Rey, I. & Kramer, F. An analysis on ensemble learning optimized medical image classification with deep convolutional neural networks. *IEEE Access* **10**, 66467–66480 (2022).

[59] Zhang, L., Ni, Q., Zhai, M., Moreno, J. & Briso, C. An ensemble learning scheme for indoor-outdoor classification based on KPIs of LTE network. *IEEE Access* **7**, 63057–63065 (2019).

[60] Jin, J. et al. A novel deep neural network topology for parametric modeling of passive microwave components. *IEEE Access* **8**, 82273–82285 (2020).

[61] Jin, J. et al. Deep neural network technique for high-dimensional microwave modeling and applications to parameter extraction of microwave filters. *IEEE Trans. Microw. Theory Techn.* **67**(10), 4140–4155 (2019).

[62] Yücel, A. C., Bağcı, H. & Michielssen, E. “An ME-PC enhanced HDMR method for efficient statistical analysis of multiconductor transmission line networks”. *IEEE Trans Comp. Packag. & Manuf. Techn.* **5**(5), 685–696 (2015).

[63] Becerra, J. A. et al. A doubly orthogonal matching pursuit algorithm for sparse predistortion of power amplifiers. *IEEE Microw. Wirel. Comp. Lett.* **28**(8), 726–728 (2018).

[64] Kennedy, M. C. & O’Hagan, A. Predicting the output from complex computer code when fast approximations are available. *Biometrika* **87**, 1–13 (2000).

[65] Wang, F. et al. “Bayesian model fusion: large-scale performance modeling of analog and mixed-signal circuits by reusing early-stage data”. *IEEE Trans Comp.-Aided Design Integr. Circuits Syst.* **35**(8), 1255–1268 (2016).

[66] Jacobs, J. P. & Koziel, S. Two-stage framework for efficient gaussian process modeling of antenna input characteristics. *IEEE Trans. Ant. Prop.* **62**(2), 706–713 (2014).

[67] Koziel, S. & Pietrenko-Dabrowska, A. *Performance-driven surrogate modeling of high-frequency structures* (Springer, 2020).

[68] Pietrenko-Dabrowska, A., Koziel, S. & Zhang, Q. J. Cost-efficient two-level modeling of microwave passives using feature-based surrogates and domain confinement. *Electronics* **12**(17), 3560 (2023).

[69] Koziel, S., Pietrenko-Dabrowska, A. & Leifsson, L. Improved efficacy behavioral modeling of microwave circuits through dimensionality reduction and fast global sensitivity analysis. *Sci. Rep.* **14**, 19465 (2024). 39174591 10.1038/s41598-024-70246-4 PMC11341873

[70] Koziel, S., Calik, N., Mahouti, P. & Belen, M. A. Accurate modeling of antenna structures by means of domain confinement and pyramidal deep neural networks. *IEEE Trans. Ant. Prop.* **70**(3), 2174–2188 (2022).

[71] Koziel, S., Pietrenko-Dabrowska, A. & Ullah, U. Reduced-cost microwave modeling using constrained domains and dimensionality reduction. *Sci. Rep.* **13**, 18509 (2023). 37898649 10.1038/s41598-023-45890-x PMC10613285

[72] Fu, J. et al. Feature-assisted neural network surrogate-based multiphysics optimization for microwave filters. *IEEE Microwave Wireless Techn. Lett.* **34**(5), 474–477 (2024).

[73] He, Y. et al. Hybrid method of artificial neural network and simulated annealing algorithm for optimizing wideband patch antennas. *IEEE Trans. Antennas Propag.* **72**(1), 944–949 (2024).

[74] Touhami, A., Collardey, S. & Sharaiha, A. A global optimization method for wideband and small supergain arrays design using artificial neural network. *IEEE Open J. Ant. Propag.* **4**, 1016–1028 (2023).

[75] Liu, Y. et al. An efficient method for antenna design based on a self-adaptive Bayesian neural network-assisted global optimization technique optimization. *IEEE Trans. Ant. Propag.* **70**(12), 11375–11388 (2022).

[76] Sonker, A., Nayak, A. K., Goel, T. & Patnaik, A. Multifunctional antenna design for wireless consumer electronic devices: a soft-computing approach. *IEEE Can. J. Electr. Comput. Eng.* **46**(2), 144–156 (2023).

[77] Y. Liu, P. Chen, J. Tian, J. Xiao, S. Noghanian, and Q. Ye, 2024 “Hybrid ANN-GA optimization method for minimizing the coupling in MIMO antennas,” *AEU – Int. J. Electronics Comm *175 155068

[78] Javid-Hosseini, S.-H., Ghazanfarianpoor, P., Nayyeri, V. & Colantonio, P. A unified neural network-based approach to nonlinear modeling and digital predistortion of RF power amplifier. *IEEE Trans. Microw. Theory Techn.* **72**(9), 5031–5038 (2024).

[79] Wu, Q., Chen, W., Yu, C., Wang, H. & Hong, W. Machine-learning-assisted optimization for antenna geometry design. *IEEE Trans. Ant. Propag.* **72**(3), 2083–2095 (2024).

[80] Yu, C., Li, Q., Feng, F. & Zhang, Q. J. Convolutional neural network with adaptive batch-size training technique for high-dimensional inverse modeling of microwave filters. *IEEE Microw. Wirels. Techn. Lett.* **33**(2), 122–125 (2023).

[81] Gupta, A., Karahan, E. A., Bhat, C., Sengupta, K. & Khankhoje, U. K. Tandem neural network based design of multiband antennas. *IEEE Trans. Ant. Propag.* **71**(8), 6308–6317 (2023).

[82] Wang, J., Fan, C., Liao, Y. & Zhou, L. Pattern-to-absorption prediction for multilayered metamaterial absorber based on deep learning. *IEEE Microwe. Wirel. Techn. Lett.* **34**(5), 463–466 (2024).

[83] Stanković, Z. Ž, Olćan, D. I., Dončov, N. S. & Kolundžija, B. M. Consensus deep neural networks for antenna design and optimization. *IEEE Trans. Ant. Propag.* **70**(7), 5015–5023 (2022).

[84] Yasmeen, K., Mishra, K. V., Subramanyam, A. V. & Ram, S. S. Circularly polarized Fabry-Pérot cavity sensing antenna design using generative model. *IEEE Sens. Lett.* **7**(2), 1–4 (2023).37529707

[85] Kouhalvandi, L. & Matekovits, L. Hyperparameter optimization of long short-term memory-based forecasting DNN for antenna modeling through stochastic methods. *IEEE Ant. Wirel. Propag. Lett.* **21**(4), 725–729 (2022).

[86] Tan, J., Shao, Y., Zhang, J. & Zhang, J. Efficient antenna modeling and optimization using multifidelity stacked neural network. *IEEE Trans. Ant. Propag.* **72**(5), 4658–4663 (2024).

[87] Li, J. et al. An adaptive evolutionary neural network-based optimization design method for wideband dual-polarized antennas. *IEEE Trans. Ant. Propag.* **71**(10), 8165–8172 (2023).

[88] G. Qi, S. Bao, Y. Wei, and Y. Zhang, “Accurate antenna design by deep auto-encoder surrogate model assisted particle swarm optimization,” *2022 IEEE 5th Int. Conf. electronic information comm. technology (ICEICT)* Hefei China 2022 875-880.

[89] Liu, J. P., Wang, B. Z., Chen, C. S. & Wang, R. Inverse design method for horn antennas based on knowledge-embedded physics-informed neural networks. *IEEE Ant. Wirel. Propag. Lett.* **23**(6), 1665–1669 (2024).

[90] Liu, Y. F., Peng, L. & Shao, W. An efficient knowledge-based artificial neural network for the design of circularly polarized 3-D-printed lens antenna. *IEEE Trans. Ant. Propag.* **70**(7), 5007–5014 (2022).

[91] Khan, M. R., Zekios, C. L., Bhardwaj, S. & Georgakopoulos, S. V. A deep learning convolutional neural network for antenna near-field prediction and surrogate modeling. *IEEE Access* **12**, 39737–39747 (2024).

[92] Su, Y. et al. Time-domain scattering parameters-based neural network inverse model for antenna designs. *IEEE Ant. Wirel. Propag. Lett.* **23**(7), 1976–1980 (2024).

[93] Jin, J., Su, Q., Xu, Y., He, Z. & Lu, Y. Efficient radiation pattern prediction of array antennas based on complex-valued graph neural networks. *IEEE Ant. Wirel. Propag. Lett.* **21**(12), 2467–2471 (2022).

[94] Tang, K., Yu, C. & Liu, Y. Cascaded neural network module for digital predistortion under various operating conditions. *IEEE Microw. Wirel. Techn. Lett.* **34**(1), 96–98 (2024).

[95] Liu, W. et al. Second-order sensitivity neural network modeling approach with applications to microwave devices. *IEEE Trans. Microw. Theory Techn.* **72**(7), 3980–3992 (2024).

[96] Faraji, A. et al. Hybrid batch-normalized deep feedforward neural network incorporating polynomial regression for high-dimensional microwave modeling. *IEEE Trans. Circuits Syst. I* **71**(3), 1245–1258 (2024).

[97] Li, X. R. & Zhao, Z. Evaluation of estimation algorithms part I: incomprehensive measures of performance. *IEEE Trans. Aerosp. Electr. Syst.* **42**(4), 1340–1358 (2006).

[98] Gorissen, D., Crombecq, K., Couckuyt, I., Dhaene, T. & Demeester, P. A surrogate modeling and adaptive sampling toolbox for computer based design. *J. Mach. Learning Res.* **11**,2051–2055(2010).

[99] J. Snoek, H. Larochelle, R.P. Adams, 2012 Practical Bayesian optimization of machine learning algorithms. *Adv. Neural Inf. Processing Syst*10.48550/arXiv.1206.2944

[100] D.P. Kingma and J. Ba, “Adam: a method for stochastic optimization,”*Int. Conf. Learning Representations (ICLR)*, Banff, AB, Canada 12–14 2014.

[101] Morris, M. D. Factorial sampling plans for preliminary computational experiments. *Technometrics* **33**, 161–174 (1991).

[102] Iooss, B. & Lemaitre, P. A review on global sensitivity analysis methods. In *Uncertainty management in simulation-optimization of complex systems* (eds Dellino, G. & Meloni, C.) 101–122 (Springer, 2015).

[103] Tian, W. A review of sensitivity analysis methods in building energy analysis. *Renew. & Sustain. Energy Rev.* **20**, 411–419 (2013).

[104] Saltelli, A. Making best use of model evaluations to compute sensitivity indices. *Com. Physics. Comm.* **145**, 280–297 (2002).

[105] Jansen, M. J. W. Analysis of variance designs for model output. *Comp. Physics Comm.* **117**, 25–43 (1999).

[106] Kovacs, I., Topa, M., Buzo, A., Rafaila, M. & Pelz, G. Comparison of sensitivity analysis methods in high-dimensional verification spaces. *Acta Tecnica Napocensis. Electr. & Telecommun.* **57**(3), 16–23 (2016).

[107] B. Beachkofski R. Grandhi, Improved distributed hypercube sampling *American Institute of Aeronautics and Astronautics* paper AIAA 2002–1274 2002

[108] Jolliffe, I. T. *Principal component analysis* 2nd edn. (Springer, 2002).

[109] Forrester, A. I. J. & Keane, A. J. Recent advances in surrogate-based optimization. *Prog. Aerospace Sci.* **45**, 50–79 (2009).

[110] C.K.I. Williams C.E. Rasmussen Gaussian processes for regression In D. Touretzky, M.C. Mozer, and M. Hasselmo (Eds) *Advances in Neural Information Processing Systems* 8 (MIT Press 1995)

[111] M. Awad R. Khanna Support vector regression. In *Efficient Learning Machines* (Apress, Berkeley, CA, 2015)

[112] Vang-Mata, R. *Multilayer perceptrons* (Nova Science Pub Inc., 2020).

[113] S. Koziel and A.T. Sigurdsson, “Performance-driven modeling of compact couplers in restricted domains *Int. J. RF & Microwave CAE*, 28 6 2018

[114] Tseng, C. & Chang, C. A rigorous design methodology for compact planar branch-line and rat-race couplers with asymmetrical T-structures. *IEEE Trans. Microw. Theory Techn.* **60**(7), 2085–2092 (2012).

[115] Lin, Z. & Chu, Q.-X. A novel approach to the design of dual-band power divider with variable power dividing ratio based on coupled-lines. *Prog. Electromagn. Res.* **103**, 271–284 (2010).

